# High-Resolution Quantification of Two-Way Nanobody Synergy Using Automated Liquid Handling and Computational Modeling

**DOI:** 10.21769/BioProtoc.5615

**Published:** 2026-03-05

**Authors:** Natasha M. Bourgeois, Satchel W. Bell, Jean Paul Olivier, Michael P. Rout, John D. Aitchison, Fred D. Mast

**Affiliations:** 1 Center for Global Infectious Disease Research, Seattle Children's Research Institute, Seattle, WA, USA; 2 Laboratory of Cellular and Structural Biology, The Rockefeller University, New York, New York, USA; 3 Department of Pediatrics, University of Washington, Seattle, WA, USA; 4 Department of Biochemistry, University of Washington, Seattle, WA, USA

**Keywords:** Single domain antibody, Nanobody, Synergy, Therapeutics, Antivirals, Automated liquid handling

## Abstract

Evaluating single-domain antibody cooperativity is essential for developing potent, escape-resistant antiviral biologics. Here, we present a protocol that reproducibly quantifies functional synergy between neutralizing nanobody pairs in standardized viral infectivity assays. Controlled automated liquid handling prepares two-dimensional concentration matrices, minimizing pipetting variance and systematic error. Neutralization data are fitted using quantitative models that independently estimate potency, cooperativity, and efficacy to distinguish additive, synergistic, and antagonistic effects between nanobody pairs. Replicated measurements enable statistically interpretable parameter estimates, supporting robust evaluation of combinatorial nanobody therapeutics with commonly available equipment and open-source analysis tools. This framework is broadly applicable to assessing cooperative effects among other classes of binding or inhibitory molecules, facilitating systematic discovery of synergistic combinations.

Key features

• Understanding functional synergistic effects between antiviral nanobodies advances their rational therapeutic design.

• Performing nanobody synergy experiments with an automated liquid handling workflow enables high-throughput and highly reproducible analysis of nanobody cooperativity.

• Evaluating a two-dimensional matrix of nanobody concentrations using the MuSyC model provides the resolution needed to understand how one nanobody can be influenced by another.

• This protocol integrates experimental, computational, and visualization procedures into a unified framework to identify and characterize synergistic antiviral nanobody combinations.

## Graphical overview


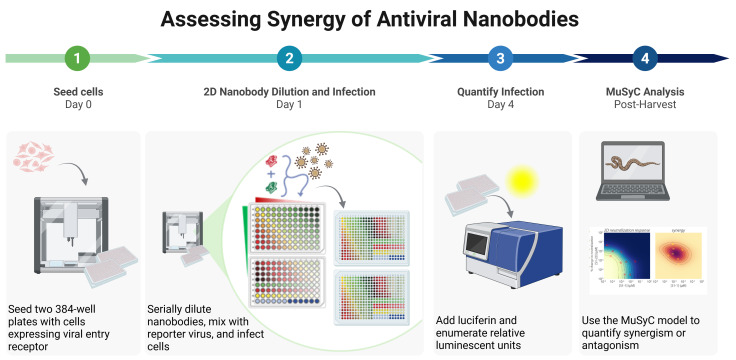


**Workflow for quantifying synergy of two nanobodies using automated liquid handling.** (1) Cells expressing viral entry receptor (e.g., HEK293T-hACE2) are seeded into two white 384-well plates. (2) Nanobodies are serially diluted either in combination or alone, then mixed with virus as shown, where red indicates nanobody 1 alone, green indicates nanobody 2 alone, yellow indicates combinations of equal concentrations of each nanobody, light blue represents the culture medium–only control, dark blue indicates the virus-only control, and the gradient from red to green represents the array of combinations of the two nanobodies. The nanobody-virus mix is incubated for 1 h and then added to seeded cells in quadruplicate across the two plates, followed by incubation for at least 56 h. (3) Relative luminescent units are quantified using a modified protocol for the Steady Glo^®^ luciferase assay system. (4) Data are analyzed in Python with the MuSyC model to evaluate synergy.

## Background

Single-domain antibodies, often referred to as nanobodies, are increasingly pursued as therapeutic agents owing to their high stability, specificity, and ease of engineering and production. The small size of nanobodies also allows for multiple nanobodies to bind a single target simultaneously, conferring resistance to mutational escape and enabling the potential for synergistic enhancement of neutralization. Synergy arises when concurrent binding to distinct epitopes produces a neutralization effect that exceeds the level predicted from the independent, additive actions of each individual nanobody. Nanobodies optimized for synergy require less material and can exhibit dramatically increased potency. Quantifying such synergy is essential for rational development of optimal nanobody combination therapies.

Classical models for synergy assessment, such as Bliss independence or Loewe additivity, assume uniform potency and maximal efficacy, and therefore often fail to capture the full pharmacodynamic behavior of nanobody pairs [1–3]. To overcome these limitations, several quantitative models have been developed to describe drug and antibody combination effects, each emphasizing different mechanistic dimensions of interaction. The synergy Python package implements a suite of quantitative models, including the Highest Single Agent (HSA), Bliss, Loewe, Zero Interaction Potency (ZIP), Bivariate Response to Additive Interacting Doses (BRAID), and Multidimensional Synergy of Combinations (MuSyC) models [4]. HSA and Bliss estimate deviations from additivity, Loewe considers self-additivity, and ZIP quantifies deviations from expected non-interaction surfaces [5]. BRAID models dose-response curvature to capture more complex nonlinear effects, while MuSyC extends the Hill equation into two dimensions to independently resolve changes in potency and maximal efficacy from the contribution of each individual neutralizing agent [5]. In this protocol, we focus on the MuSyC model because of its ability to separate potency and efficacy effects, which is critical for interpreting nanobody effects, while maintaining compatibility with alternative frameworks within the same computational environment. This modularity, combined with robust automated preparation of 2D concentration matrices, enables users to evaluate their datasets under multiple synergy definitions and select the most biologically appropriate model for their system.

Despite the availability of multiple synergy models, a high-throughput, complete, and standardized methodology for generating comprehensive two-dimensional concentration matrices required for robust model fitting is not currently available. Here, we describe an integrated and standardized experimental and computational pipeline for quantitative 2D synergy testing using nanobody pairs. Automated liquid handling using the Gilson PipetteMax liquid handling robot ensures accurate and reproducible preparation of dose–response matrices, while standardized pseudoviral neutralization assays provide consistent biological readouts. Data are analyzed with the synergy Python package to estimate potency, cooperativity, and efficacy parameters across MuSyC, BRAID, and related models. Custom Python scripts visualize the data and assist in interpreting the cooperative effects of nanobody pairs. Together, these elements establish a reproducible, adaptable pipeline that links controlled experimental design with transparent computational analysis, enabling rigorous and transferable assessment of cooperative interactions among nanobody therapeutics and other biologic combinations.

## Materials and reagents

**Biological materials**

*Notes:*

*1. The following materials can be obtained from our lab or generated independently. See the references for methodology.*

*2. See General note 9 for information on storage shelf-life of biological materials, reagents, and solutions.*

1. Nanobodies of interest, purified and enumerated, stored in 20 mM HEPES, pH 7.4, 150 mM NaCl, at 4 °C [6]

2. Titrated lentiviral pseudovirus (PSV) expressing luciferase reporter, stored at -70 °C; for example, viral spike protein (such as Addgene #194494) and firefly luciferase 4-Luc (Addgene #21725) packaged with psPAX2 (Addgene #12260) [6]

3. Viral entry receptor-expressing HEK293T17 cells, stored at -196 °C [6]

**Reagents**

1. DMEM (Gibco, catalog number: 11965-092), store at 4 °C

2. HEPES (Gibco, catalog number: 15630-080), store at 4 °C

3. FBS (Biowest, catalog number: S1620), store at -20 °C

4. Steady-Glo^®^ luciferase assay system (Promega, catalog number: E2510), store at -20 °C

5. Bleach (Clorox), storage at RT

6. Trypan Blue (Corning, catalog number: 25-900-Cl), store at room temperature

7. 0.05% trypsin–EDTA (Gibco, catalog number: 25300-054), store at 4 °C

8. Dimethyl sulfoxide (DMSO) (Sigma-Aldrich, catalog number: D8418), store at room temperature

**Solutions**

1. Complete medium (see Recipes)

2. Freezing medium (see Recipes)

**Recipes**

**1. Complete medium**

**Table:** 

Reagent	Final concentration	Quantity or volume
DMEM	n/a	500 mL
FBS	10% v/v	50 mL
HEPES	25 mM	12.5 mL

Store at 4 °C; shelf-life: 1 month.

**2. Freezing medium**

**Table:** 

Reagent	Final concentration	Quantity or volume
Complete medium	90% v/v	9 mL
DMSO	10% v/v	1 mL

Store at 4 °C; shelf-life: 1 month.

**Laboratory supplies**

1. 12-channel reservoir (Axygen, catalog number: RES-MW12-HP-SI)

2. 384-well white-bottom assay plates (Falcon, catalog number: 353988)

3. 96-well V-bottom assay plates (Corning Costar, catalog number: 3894)

4. 96-well deep-well plates (Biotix, catalog number: 63300108)

5. DF200 Pipetman tips (Gilson, catalog number: F172503)

6. Beaker for bleach (Nalgene, catalog number: 1201)

## Equipment

1. Gilson PipetteMax automated liquid handler (Gilson, catalog number: 32100000)

2. Gilson MAX8x200 pipette head (Gilson, catalog number: LC12001)

3. Biosafety cabinet (NUAIRE, catalog number: NU-543)

4. CO_2_ incubator (Thermo Scientific, model: Series 8000 WJ, catalog number: 3578)

5. Automated cell counter (Bio-Rad, catalog number: TC20)

6. Luminescence plate reader (Molecular Devices, model: Spectramax i3x)

7. Computer (64 GB RAM suggested for sufficient headroom for robust, parallelized analysis of large synergy datasets, but smaller configurations are adequate for simpler analyses)

## Software and datasets

**Table:** 

Type	Software/dataset/resource	Version	Date	License	Access (free or paid)
Software	Microsoft Excel for Microsoft 365	2502	March 11, 2025	Proprietary commercial	Paid
Software	python	3.10.14	March 19, 2024	-	Free
Software	synergy	0.5.1	June 29, 2021	-	Free
Software	matplotlib	3.9.1	July 4, 2024	-	Free
Software	seaborn	0.13.2	January 25, 2024	-	Free
Software	plotly	5.22.0	May 1, 2024	-	Free
Software	numpy	2.0.0	June 16, 2024	-	Free
Software	pandas	2.2.2	April 10, 2024	-	Free
Software	scipy	1.14.0	June 24, 2024	-	Free
Software	openpyxl	3.1.4	May 29, 2024	-	Free
Software	ipykernel	6.30.1	August 4, 2025	-	Free
Software	nbformat	5.10.4	April 4, 2024	-	Free
Software	kaleido	1.0.0	June 19, 2025	-	Free
Software	miniforge3	25.3.0	April 5, 2025	-	Free
Software	Visual Studio Code	1.103.2	August 7, 2025	-	Free
Software	TRILUTION^®^ micro for the Gilson PIPETMAX^®^	3.0	May 1, 2018	Included with hardware purchase	Paid
Software	Protocol Builder for the Gilson PIPETMAX^®^	2.1		Included with hardware purchase	Paid

*Note: This protocol uses Microsoft Windows throughout. However, other operating systems and software applications can also be used.*

## Procedure

**Figure 1.**
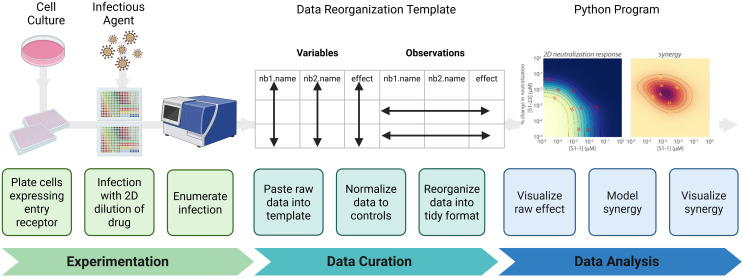
Synergy protocol workflow. (Experimentation) Infectious agent-permissive cells are plated and infected with an agent in the presence of a two-dimensional dilution series of the drug; then, infection is enumerated. (Data Curation) Data are transferred into a standardized template to avoid copy errors. Data are normalized to appropriate controls and then reformatted into a tidy structure suitable for downstream analysis. (Data analysis) A Python-based analysis pipeline is used to visualize raw neutralization effects, mathematically model the relationship between the two drugs tested, and visualize if the relationship is antagonistic, additive, or synergistic.

The protocol described herein provides detailed instructions specific to the indicated materials and reagents. However, the workflow may be readily adapted to alternative experimental settings and data analysis pipelines. Figure 1 outlines the key steps of the protocol that can be extended to additional contexts.

**A. Cell passage and plating**

1. Collect HEK293T17 cells expressing the viral entry receptor into a conical tube.

**Critical:** Cells can be maintained as a continuously cultured line in complete medium or prepared in advance as frozen, ready-to-use aliquots. For long-term storage, freeze cells in complete growth medium containing 10% (v/v) DMSO at -80 °C or in liquid nitrogen. To prepare aliquots, expand cells to ~80% confluence, detach with 0.05% trypsin–EDTA, resuspend in freezing medium, and dispense 1 × 10^6^ cells/mL into cryovials. Rapidly freeze using a controlled-rate freezing container (1 °C/min) before transferring to long-term storage. When needed, thaw one aliquot quickly at 37 °C, dilute immediately into prewarmed complete medium, and culture for at least one passage to ensure >90% viability before use.

2. Centrifuge in a conical tube at 230× *g* for 3 min at room temperature.

3. Carefully aspirate the supernatant so as not to disturb the cell pellet and then resuspend the pellet in 10 mL of prewarmed (37 °C) complete medium.

4. Mix a 10 μL aliquot of the cell suspension with 10 μL of Trypan Blue solution to prepare a 1:1 solution. Count viable cells using a Bio-Rad TC20 Automated Cell Counter or a standard hemocytometer. Record both total and viable cell numbers.

**Critical:** Ensure viability is >90% before proceeding; low-viability cultures produce uneven seeding and variable infection efficiency.

*Note: Alternative cell lines may be used according to your experimental needs. Follow the storage and maintenance procedures provided by the source of your cell line if using a cell line other than HEK293T17.*

**Figure 2.**
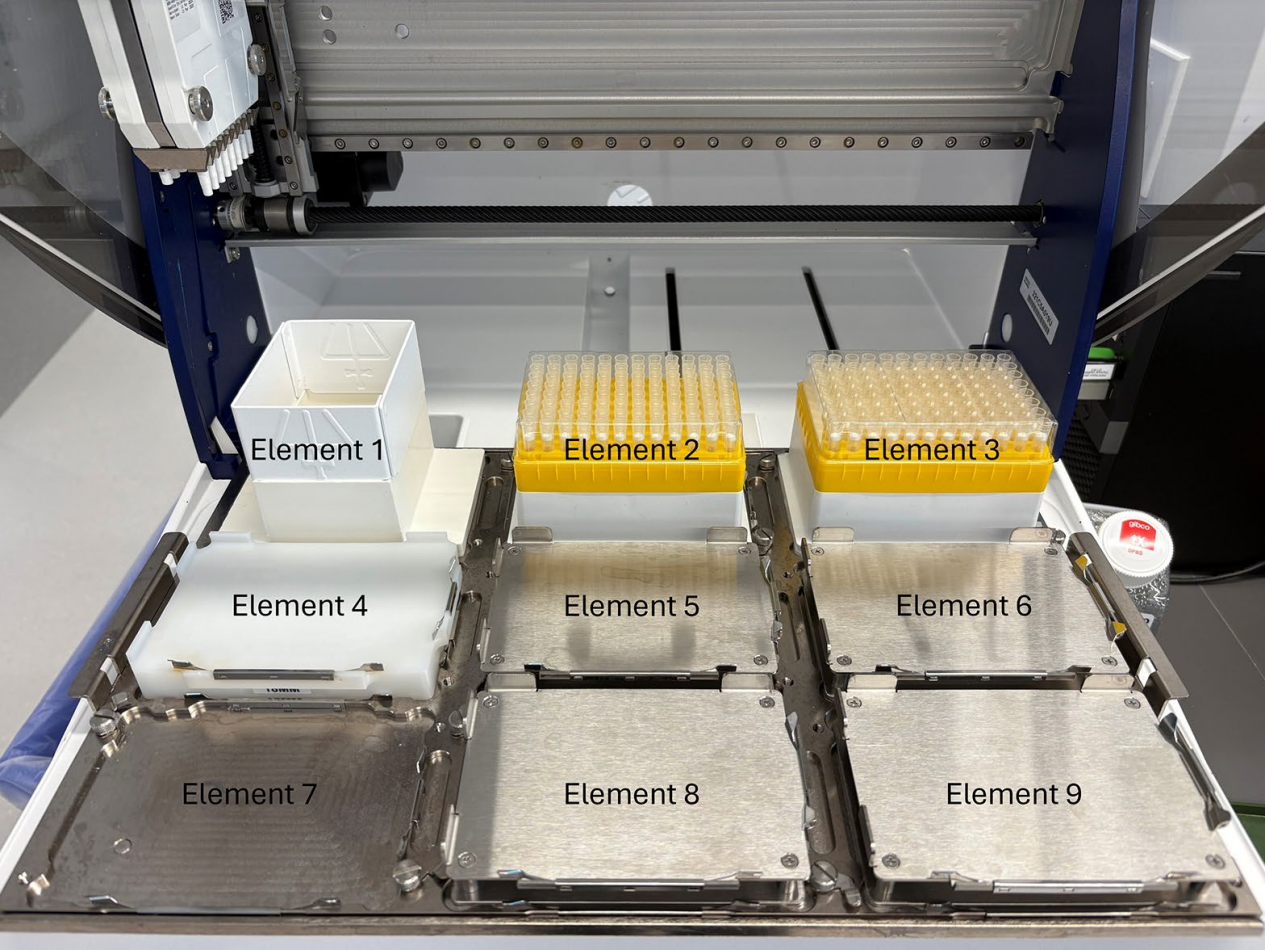
Gilson PipetteMax deck layout and element nomenclature used throughout this protocol. View of the Gilson automated liquid handler with elements numbered according to TRILUTION^®^ micro software conventions. Element 1 = tip waste. Elements 2 and 3 = tip racks. Elements 4–9 = plate positions. Orientation is shown from the front of the instrument (user side) toward the back. These element designations are referenced in the protocol for all automated steps (e.g., cell plating, nanobody dilution, and virus addition).

**Figure 3.**
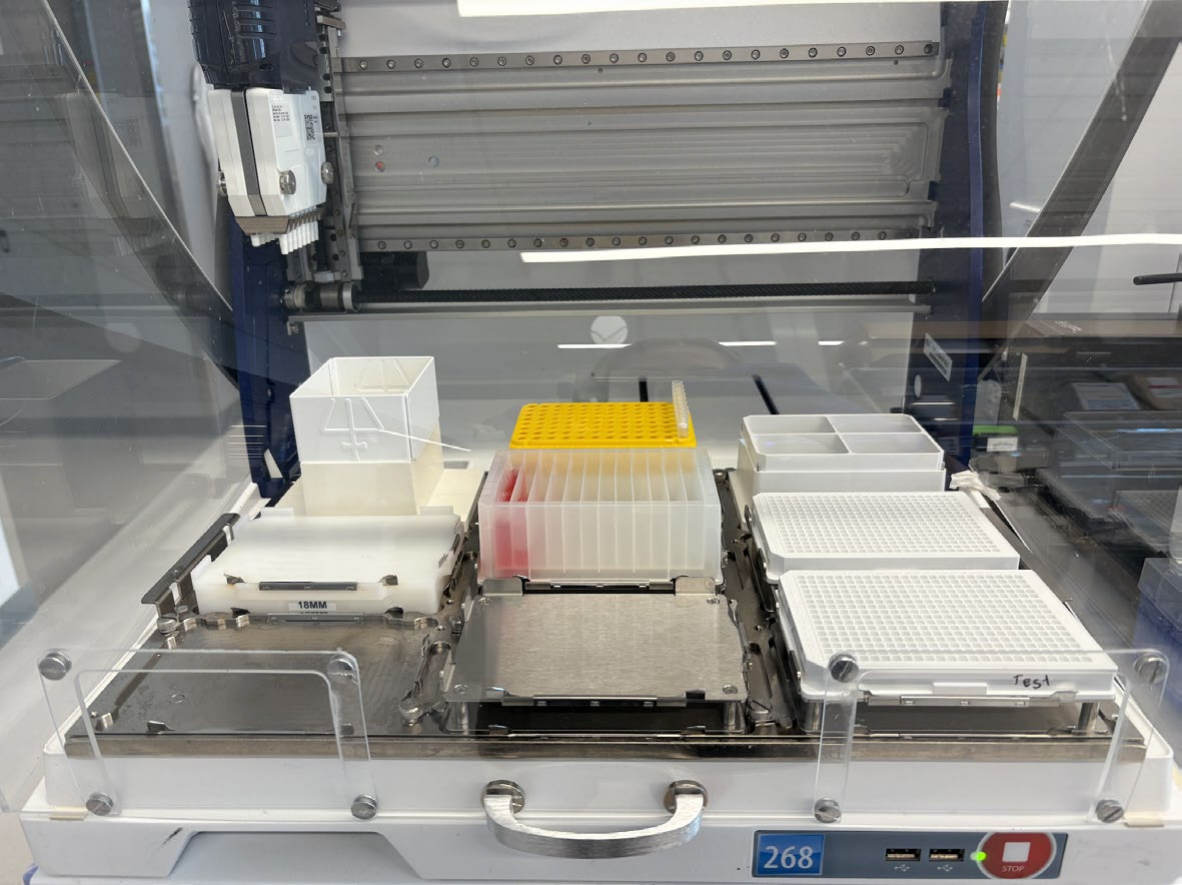
Gilson PipetteMax deck configuration for the cell plating protocol (Supplementary File 1). Shown is the Gilson automated liquid handler arranged for automated cell plating. A 12-channel reagent reservoir is placed in Element 5, containing a suspension of 2 × 10^5^ cells/mL distributed as 15 mL in the first (left-most) channel and 5 mL in the second channel. Sterile, white 384-well assay plates (with lids removed) are placed in Elements 6 and 9. Tip racks are loaded in Element 2 (positions A-H12).

5. Using the viable cell count, prepare 20 mL of cell suspension in complete medium at 2 × 10^5^ cells/mL for each nanobody pair to be tested. Mix gently by inversion or slow pipetting to maintain a uniform suspension and prevent cell clumping.

6. Using the Cell Plating protocol (Supplementary File 1) on a Gilson PipetteMax automated liquid dispenser (hereafter referred to as “Gilson”), distribute 20 μL of the prepared cell suspension into each well of two sterile, white-bottom 384-well assay plates. Labware placement locations on the Gilson deck are shown in Figure 2. Figure 3 shows the Gilson deck fully configured to run this cell plating protocol.

7. Setup of the Gilson deck:

a. Place a 12-channel reservoir in Element 5 of the Gilson.

b. Load tips in Element 2. This protocol uses tips A-H12 from Element 2.

c. Load sterile, white 384-well plates in Element 6 and in Element 9. Remove their lids.

d. Transfer 15 mL of the 2 × 10^5^ cells/mL suspension into the first (left-most) channel and 5 mL of cell solution into the second channel of the 12-channel reservoir.

**Figure 4.**
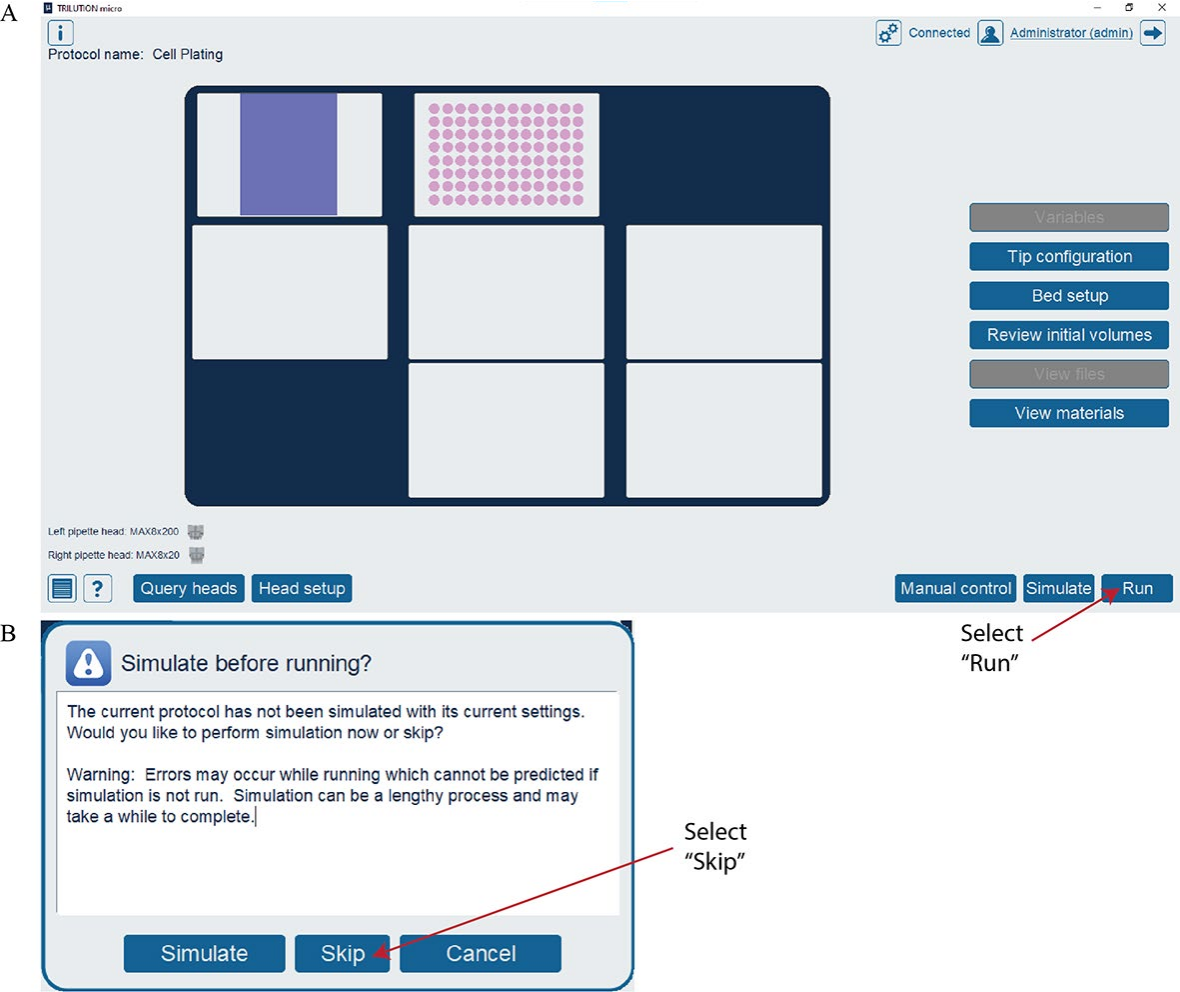
Running the Cell Plating protocol in TRILUTION^®^ micro. (A) Screenshot of the TRILUTION^®^ micro software interface after opening a protocol file. Select the *Run* button to run the protocol. (B) Screenshot of the pop-up window that may appear the first time a protocol is run. Select the *Skip* button to run the protocol.

**Critical:** Confirm that all lids are removed, liquid levels are correct, and the Cell Plating Protocol file is correctly loaded in TRILUTION^®^ micro. Verify that the Gilson is calibrated for 20 μL delivery to avoid systematic seeding variation.

e. Open the Cell Plating protocol file in TRILUTION^®^ micro and select *Run* (Figure 4A).

*Notes:*

*1. If a protocol is being run for the first time, a pop-up window may appear (Figure 4B). Select* Skip *to run the protocol.*

*2. This protocol takes 13 min and 28 s to run.*

*3. See General note 5 for important considerations on running protocols using the Gilson, including an overview of critical automation protocol parameters.*

8. Replace the lids on the 384-well plates and then incubate the cells overnight at 37 °C in a humidified incubator with 5% CO_2_.

**Critical:** Ensure plates remain level and undisturbed during incubation to promote uniform cell attachment and prevent edge-effect drying.

**Timing:** Incubate for 16–18 h or until cells reach ~70% confluence.

**Pause point:** Plates will remain in the incubator overnight and be used the following day for nanobody–virus infection.

*Note: The 12-channel reservoir can be retained to be further utilized in later steps of this protocol if kept sterile.*

**B. 2D-nanobody dilution and viral infection**

1. Prepare 10 μM working solutions of each nanobody by diluting them individually in complete medium.

a. Determine the total volume of 10 μM nanobody solution to be prepared for each nanobody. A total of 87.5 and 95 μL of nanobodies 1 and 2 are needed, respectively, at 10 μM each. Prepare a solution with at least 10 μL in excess to account for pipetting loss.

b. Calculate the volume of stock nanobody needed for the solution by using C_1_V_1_ = C_2_V_2_, where C_1_ is the stock concentration of nanobody, V_1_ is the volume of stock nanobody needed, C_2_ is the final concentration of nanobody solution needed (10 μM), and V_2_ is the total volume of 10 μM solution needed.

c. Calculate the volume of medium needed for the solution by subtracting the volume of stock nanobody (V_1_) from the final volume (V_2_).

*Note: If performing multiple synergy assays with a given nanobody, prepare one 10 μM solution sufficient for all assays.*

**Figure 5.**
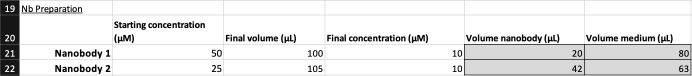
Screenshot of the Deep-Well Plate Map spreadsheet (Supplementary File 2) showing nanobody preparation calculations. Example calculation of nanobody dilution using user-specified starting concentrations and desired final concentration (10 μM). The spreadsheet automatically computes the required volume nanobody (μL) and volume medium (μL) based on the entered starting concentration and total desired volume. Users should replace the example concentrations shown for Nanobody 1 and Nanobody 2 with their own stock concentrations to populate the corresponding volume fields.

d. Use the Deep-Well Plate Map spreadsheet (Supplementary File 2) to automatically compute the volume of nanobody and complete medium needed to prepare the solution (Figure 5). Replace the example values in the “Starting concentration (μM)” column with the actual nanobody stock concentrations to populate the “Volume nanobody (μL)” and “Volume medium (μL)” columns.

**Caution:** Avoid introducing air bubbles by ensuring the pipette tip remains submerged in the solution during mixing. If required, increase the “Final volume (μL)” to ensure even mixing and to reduce relative error.

e. Pipette the calculated volumes of nanobody and complete medium into separate sterile microcentrifuge tubes. Mix gently by pipetting.

**Critical:** Do not vortex nanobody solutions. Although nanobodies are generally stable, mechanical shear from vortex mixing can promote partial unfolding or aggregation. Mix solutions gently by pipetting to maintain conformational integrity and prevent loss of activity.

2. Prepare pseudovirus solution by diluting the stock in complete medium to achieve ~1 × 10^5^ relative luciferase units (RLUs) per well.

**Figure 6.**
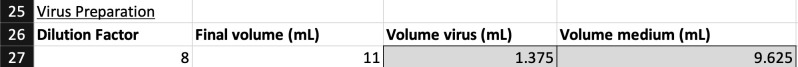
Screenshot of the Deep-Well Plate Map spreadsheet (Supplementary File 2) showing the pseudovirus preparation calculation. Example calculation for preparing a diluted pseudovirus working solution at the desired dilution factor. The spreadsheet automatically computes the required volume virus (mL) and volume medium (mL) from the user-entered dilution factor and final volume (mL) fields. Replace the example dilution factor with the experimentally determined value to generate the appropriate volumes for the assay.

**Critical:** Use only pseudovirus stock that has been previously titrated to determine its infectious dose range. Using previously titrated pseudovirus ensures that the infectious dose added to each well is within the dynamic range of the assay. The selected inoculum must yield a signal that is both well above background and within the linear dynamic range of the assay/detector, enabling reliable statistical comparison across conditions. In practice, this requires choosing a virus dilution that produces a signal at least ~2 log_10_ (≈100-fold) above background while remaining below saturation. Unquantified or variably concentrated virus stocks can produce signal saturation or undetectable infection, leading to nonlinear dose–response behavior and inaccurate synergy estimation. We have empirically found that pre-determining the dilution that yields ~1 × 10^5^ RLUs per well standardizes infection efficiency across experiments and allows direct comparison between nanobody pairs.

a. Using a prior titration curve, identify the dilution factor that yields ~1 × 10^5^ RLUs per well, accounting for the fact that equal volumes of pseudovirus and nanobody solutions will be combined before being added to cells. This results in an additional 2-fold dilution beyond the initial preparation. For example, if an 8-fold dilution of pseudoviral stock produced ~1 × 10^5^ RLUs in the titration assay, prepare the working pseudovirus solution at a 4-fold dilution to account for the additional 2-fold dilution that will occur upon adding the pseudovirus and nanobodies mixture to cells.

b. Use the Deep-Well Plate Map spreadsheet (Supplementary File 2) to automatically calculate the required volumes of pseudovirus and complete medium to prepare pseudovirus solution with 2 mL excess (Figure 6). Replace the example “Dilution Factor” column value with the experimentally determined value. The spreadsheet will then populate the “Volume virus (mL)” and “Volume medium (mL).” If less or more excess volume is needed, adjust the “Final volume (mL)” column value accordingly. When performing multiple synergy assays with the same pseudovirus stock, increase the total volume to accommodate all assays in a single preparation.

c. Pipette the calculated volumes of pseudovirus and culture medium into a conical tube.

**Figure 7.**
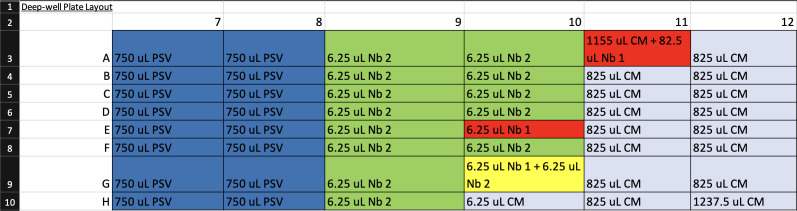
Screenshot of the Deep-Well Plate Map spreadsheet (Supplementary File 2) showing the pipetting layout for preparation of the 96-well deep-well plate. Each cell indicates the reagent and volume to be added to the corresponding well. Color coding corresponds to Step 2 of the Graphical abstract: dark blue = pseudovirus (PSV), light blue = complete medium, red = nanobody 1 (Nb 1), green = nanobody 2 (Nb 2), and yellow = 1:1 mixture of nanobodies 1 and 2. This layout guides reagent placement during preparation to ensure correct mapping for automated serial dilutions and virus–nanobody mixing on the Gilson.

3. In a biosafety cabinet equipped with micropipettes and a 10% bleach solution for decontamination, use aseptic technique to prepare a sterile 96-well deep-well plate according to the Deep-Well Plate Layout file (Figure 7, Supplementary File 2).

**Critical:** Use the rightmost columns of the deep-well plate to ensure compatibility with subsequent automated transfer steps on the Gilson and to enable reuse of the plate in subsequent assays (see General note 6).

a. Pipette 1,237.5 μL of complete medium into H12.

b. Pipette 1,155 μL of complete medium into A11.

c. Pipette 825 μL of complete medium into B–H11 and A–G12.

d. Pipette 750 μL of prepared diluted pseudovirus into A–H of columns 7 and 8.

e. Pipette 82.5 μL of prepared 10 μM nanobody 1 solution into A11.

f. Pipette 6.25 μL of complete medium into H10.

*Note: When distributing the 6.25 μL volumes, ensure that the pipette tip is centered and lightly pressed against the bottom of the well before dispensing. This ensures the small volume is accessible to the Gilson.*

g. Pipette 6.25 μL of prepared 10 μM nanobody 1 solution into E10 and G10.

h. Pipette 6.25 μL of prepared 10 μM nanobody 2 solution into A–H9, A–D10, and F–G10.

**Critical:** Dispense all 6.25 μL volumes last to minimize the effects of evaporation (see also General note 1). Work quickly.

**Figure 8.**
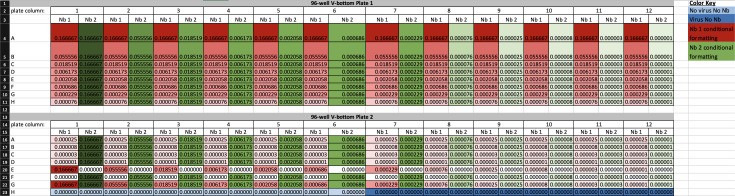
Screenshot of the 96-well V-bottom Plates Map spreadsheet (Supplementary File 4) showing final nanobody concentrations generated by the nanobody-virus mix protocol. The spreadsheet displays the calculated concentrations of nanobody 1 (Nb 1) and nanobody 2 (Nb 2) for each well of plates 1 and 2. Three-color conditional formatting highlights concentration gradients: red = Nb 1, green = Nb 2, with darker shades indicating higher concentrations. White cells denote zero concentration, while dark blue and light blue indicate virus-only and medium-only controls, respectively. This layout defines the full 2D nanobody-pair concentration matrix used for infection assays, ensuring reproducibility across experimental replicates.

**Figure 9.**
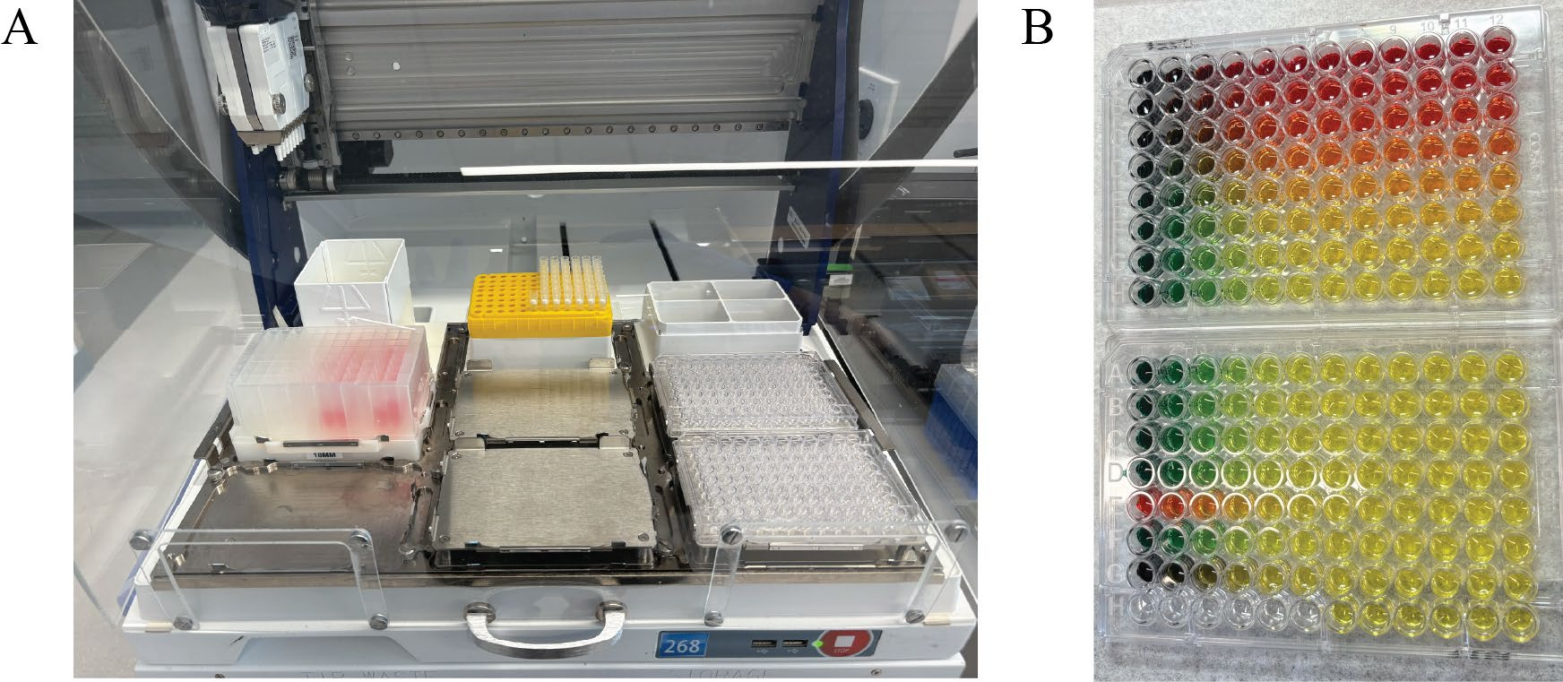
Gilson PipetteMax deck configuration and visual output of the Nanobody-Virus Mix protocol (Supplementary File 3). (A) Picture of the Gilson PipetteMax deck configured to run the Nanobody Virus Mix protocol. The prepared deep-well 96-well plate is placed in Element 4, pipette tips are loaded on Element 2, and sterile 96-well V-bottom plates are placed in Element 6 and 9. (B) Representative 96-well V-bottom plates after completion of the Nanobody-Virus Mix protocol using food dyes to visualize reagent distribution: red = nanobody 1, green = nanobody 2, yellow = nanobody mixing, and also pseudovirus alone, and no food dye (clear) = complete medium. The resulting 2D gradient demonstrates correct serial dilution and mixing across wells.

4. Run the Nanobody-Virus Mix protocol (Supplementary File 3) on the Gilson. This protocol performs 2D serial dilutions of nanobody pairs and transfers pseudovirus, resulting in two full 96-well V-bottom plates containing the nanobody-virus combinations. The 96-well V-bottom Plates Map spreadsheet (Supplementary File 4) shows the resulting contents in each well, including the exact concentration of each nanobody (Figure 8). Figure 9 shows (A) the Gilson deck setup when ready to run this protocol and (B) representative 96-well V-bottom plates prepared using food dye for visualization.

*Note: Screenshots of large datasheets are intended only to provide a reference for how the file or section appears once opened. Please use the indicated supplementary files to view details.*

a. Bring the prepared deep-well plate and two sterile 96-well V-bottom plates to the Gilson.

b. Place the deep-well plate onto Element 4 and the 96-well V-bottom plates onto Elements 6 and 9 (see General note 7).

*Note: Label the V-bottom plates with distinct identifiers, as their order must be preserved in later steps. The plate positioned farthest from the user in the Gilson is “Plate 1,” and the plate closest to the user is “Plate 2.”*

c. Load pipette tips into Element 2. This protocol uses tips 7H and 8-12A-H.

d. Ensure lids are removed from all plates and tips. Open the Nanobody Virus Mix protocol file in TRILUTION^®^ micro and select *Run*.

**Timing:** The program requires 33 min and 41 s to complete. Each well will contain 50 μL of nanobody (or medium control) and 50 μL of pseudovirus (or medium control).

**Critical:** Verify that the deck layout matches Figure 9A before initiating the run and that the plates are seated flat on the deck; misaligned labware will disrupt serial dilutions and invalidate concentration mapping.

**Figure 10.**
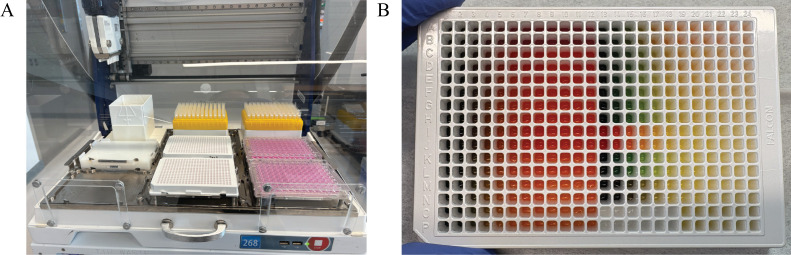
Gilson PipetteMax deck configuration and outcome of the Transfer to Cells protocol (Supplementary File 5). (A) The Gilson PipetteMax configured for the Transfer to Cells protocol. Seeded 384-well plates from step A6 are positioned in Elements 5 and 8. Sterile 96-well V-bottom plates are placed in Elements 6 and 9 in the same order as in step B4. Full tip racks are loaded into Elements 2 and 3. (B) A representative 384-well plate following completion of the protocol using the food dye 96-well V-bottom plates shown in Figure 9.

5. Incubate the two resulting 96-well V-bottom plates at 37 °C with 5% CO_2_ for 1 h.

6. Run the Transfer to Cells protocol (Supplementary File 5) on the Gilson. This protocol transfers each well of the 96-well V-bottom plates onto the 384-well plates containing cells in quadruplicate (two replicates per plate). Figure 10 shows (A) the Gilson deck setup for this protocol and (B) representative 384-well plates when this protocol is run with food dye.

a. Return the 96-well V-bottom plates to the same element positions used in step B4.

b. Place the seeded 384-well cell plates onto Elements 5 and 8.

c. Load full tip boxes into Elements 2 and 3.

d. Remove all lids from plates and tip racks. Open the Transfer to Cells protocol file in TRILUTION^®^ micro and select *Run*.

**Timing:** The protocol requires 19 min and 46 s to complete. Each well of the 384-well cell plates receives 18 μL of nanobody-virus mix or corresponding negative and positive controls.

**Figure 11.**
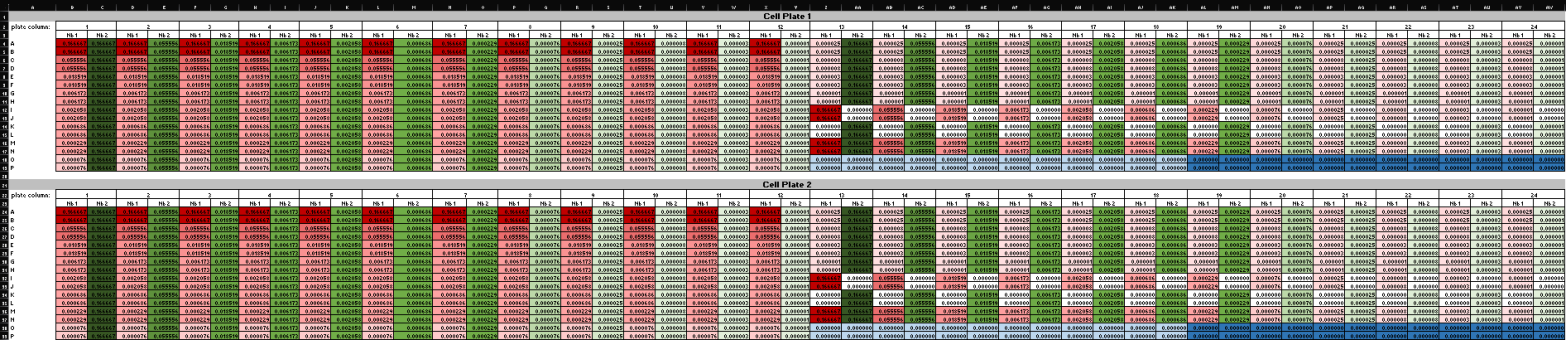
Screenshot of the Cell Plates Map spreadsheet (Supplementary File 6) showing final nanobody concentrations in each well of the 384-well cell plates. Conditional formatting follows the same color scheme used in Figure 8: red = nanobody 1 (Nb 1), green = nanobody 2 (Nb 2), white = zero concentration, dark blue = virus-only control, and light blue = medium-only control. The two 384-well plates shown are identical replicates, confirming uniform transfer during the Transfer to Cells protocol.

The Cell Plates Map spreadsheet shows the resulting contents of each well, including the concentration of each nanobody (Supplementary File 6 and Figure 11).

7. Return cell plates to 37 °C and 5% CO_2_ for at least 56 h.

**Pause point:** Plates may be left incubating at this stage for up to 72 h before proceeding to the next step.

**C. Quantifying viral infection with Steady-Glo^®^**

1. Prepare the Steady-Glo^®^ reagent in advance. Prior to the harvest day, bring Steady-Glo^®^ liquid substrate (#E254A) to 37 °C. Then, mix the entire content with powder substrate (#E253A). After dispensing 10 mL per tube aliquots, store the solution at -20 °C overnight.

2. On the day of harvest, thaw the appropriate number of aliquots (1 per 384-well plate) to bring the prepared Steady-Glo^®^ solution to 37 °C.

3. Run the Steady Glo protocol (Supplementary File 7) on the Gilson to distribute 40 μL of reagent into each well of the 384-well cell plates containing experimental solution. The initial deck configuration is identical to that shown in step A6 (Figure 3).

a. Place a 12-channel reservoir in the Element 5 position.

b. Place 384-well plates in Element 6 and in Element 9 positions.

c. Load pipette tips in Element 2. This protocol uses pipette tips A-H12 from Element 2.

d. Transfer 15 mL of warmed, prepared Steady-Glo^®^ solution into the first (leftmost) and second channels and add 5 mL into the third channel of the 12-channel reservoir.

e. Ensure lids are removed from all plates and pipette tip racks. Open the Steady Glo protocol in TRILUTION^®^ micro and select *Run*.

**Timing:** This protocol takes 12 min and 39 s to run.

4. Incubate the plates at room temperature in the dark for 15 min.

**Critical:** Wells will be filled to their maximum volume. Wipe any condensation from the plate lids before replacement and avoid bumping or shaking the plates to prevent cross-well contamination.

5. Quantify luminescence. Read the plates using a luminescent plate reader (see General note 3). Export the raw luminescent data in plate format for downstream analysis.

**Pause point:** The experimental phase concludes here. All subsequent steps involve computational analysis of the acquired data.

## Data analysis

**Figure 12.**
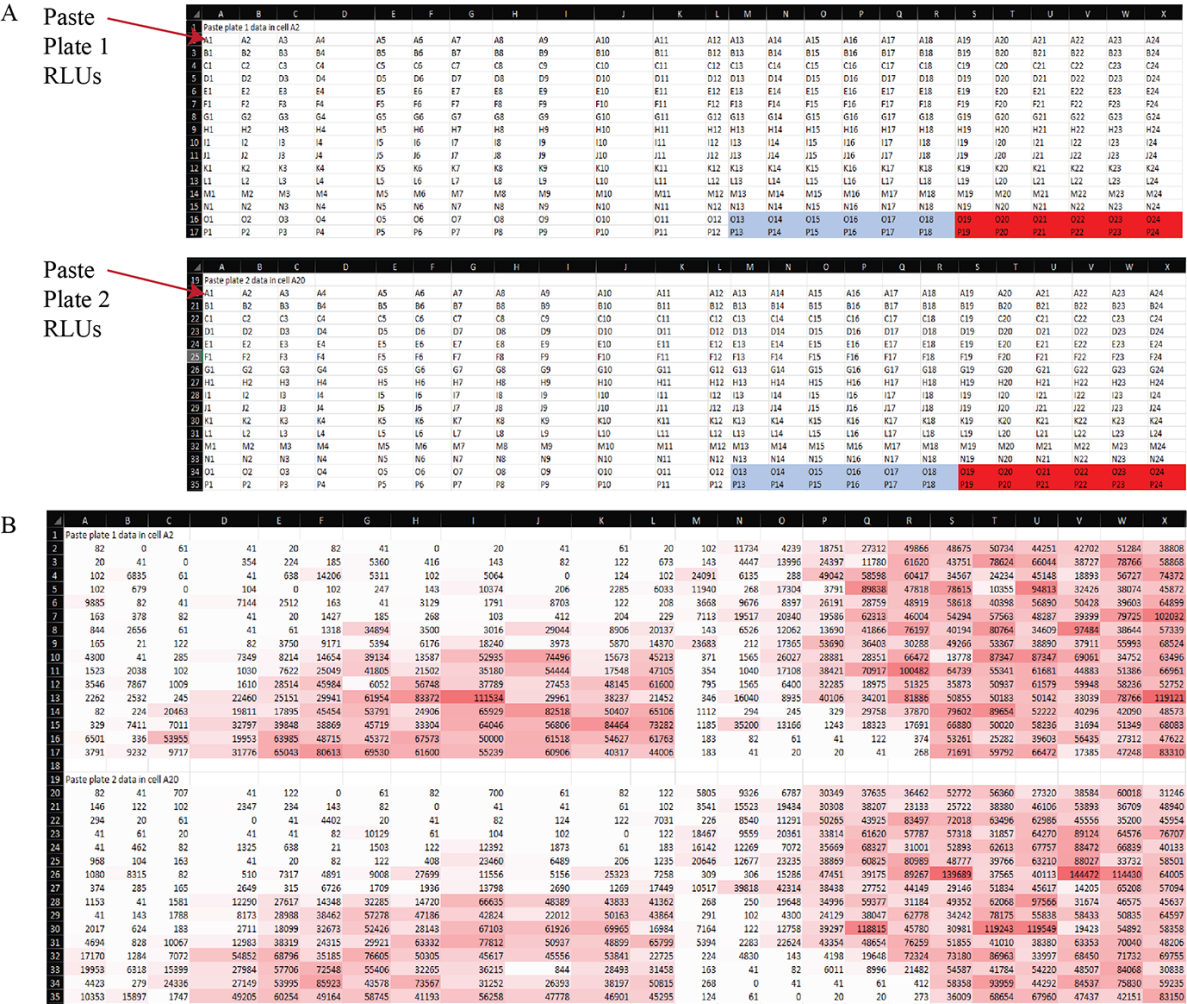
Organization of raw luminescence data for analysis in the Synergy Data Reorganization spreadsheet (Supplementary File 8). (A) Layout of the Data Reorganization sheet showing the designated input fields for raw RLU values from each 384-well plate of the synergy experiment. Raw data from plate 1 are pasted beginning in cell A2, and data from plate 2 in cell A20. Pasting values in these positions triggers automatic data restructuring into a tidy format. Light blue = negative control values (cell growth medium alone). Red = positive control values (virus only). (B) Example data copied into the Data sheet, demonstrating how the template appears after pasting raw RLU output. Conditional formatting highlights variation in luminescence intensity across wells, enabling immediate visual inspection of dynamic range and plate consistency.

1. Prepare data for analysis using the Synergy Data Reorganization spreadsheet (Supplementary File 8) on a computer equipped with Microsoft Excel.

a. For each synergy experiment, copy and paste the exported raw RLU data from plate 1 into cell A2 and from plate 2 into cell A20 (Figure 12) of the Data Reorganization sheet of Supplementary File 8.

**Figure 13.**
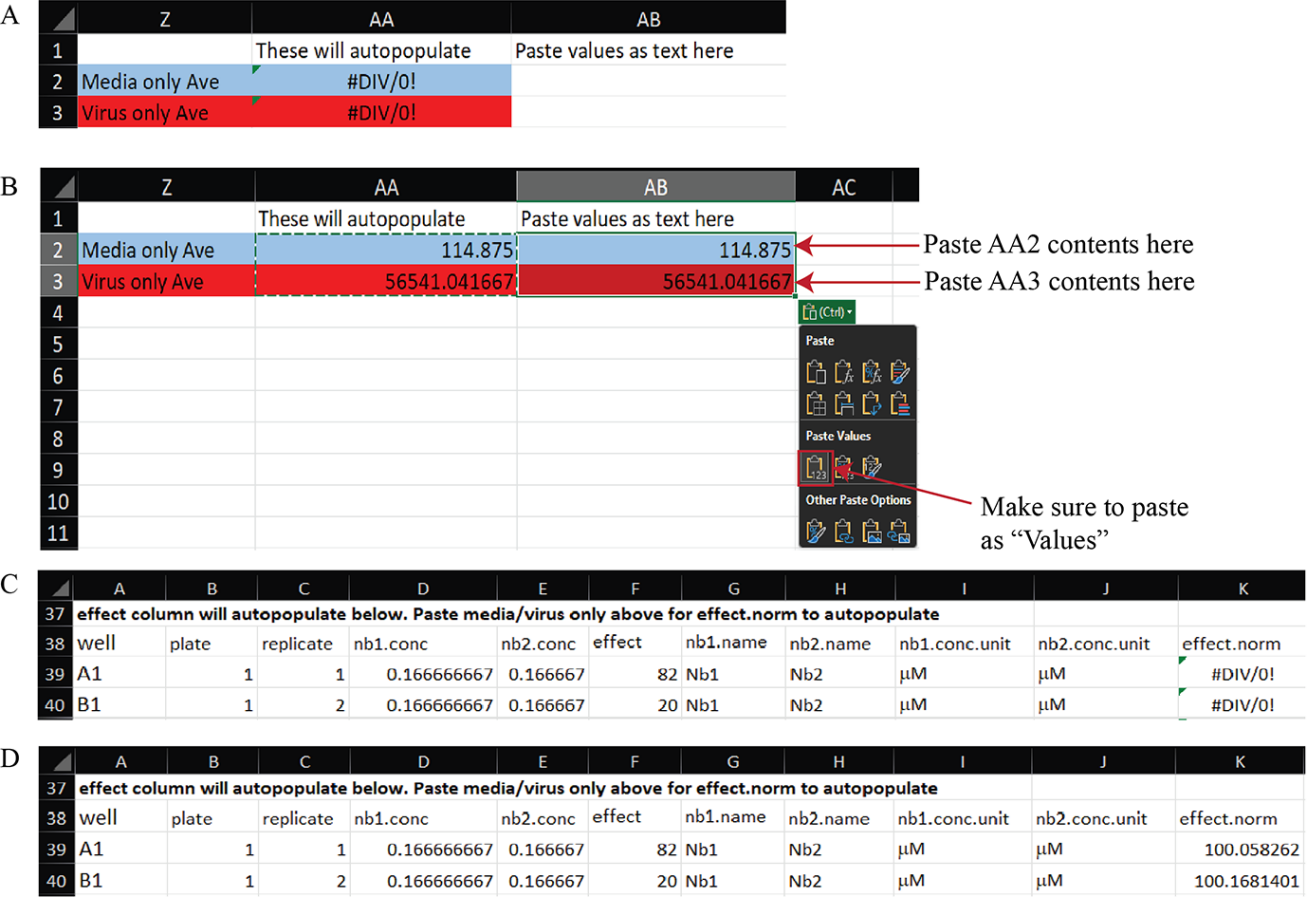
Normalization of raw luminescence data to media-only and virus-only controls in the Synergy Data Reorganization spreadsheet (Supplementary File 8). (A) A blank Data Reorganization sheet showing template fields for data entry. (B) Example Data Reorganization sheet after pasting raw RLU data and transferring control averages from cells AA2–AA3 into AB2–AB3 as values. Red and blue shading correspond to virus-only and medium-only controls, respectively. (C) Example tidy data before control values are pasted, in which the effect.norm column displays “#DIV/0!” because normalization factors are not yet defined. (D) Example tidy data after control values are pasted, showing automatic population of the effect.norm column with normalized effect values representing percent neutralization.

**Figure 14.**
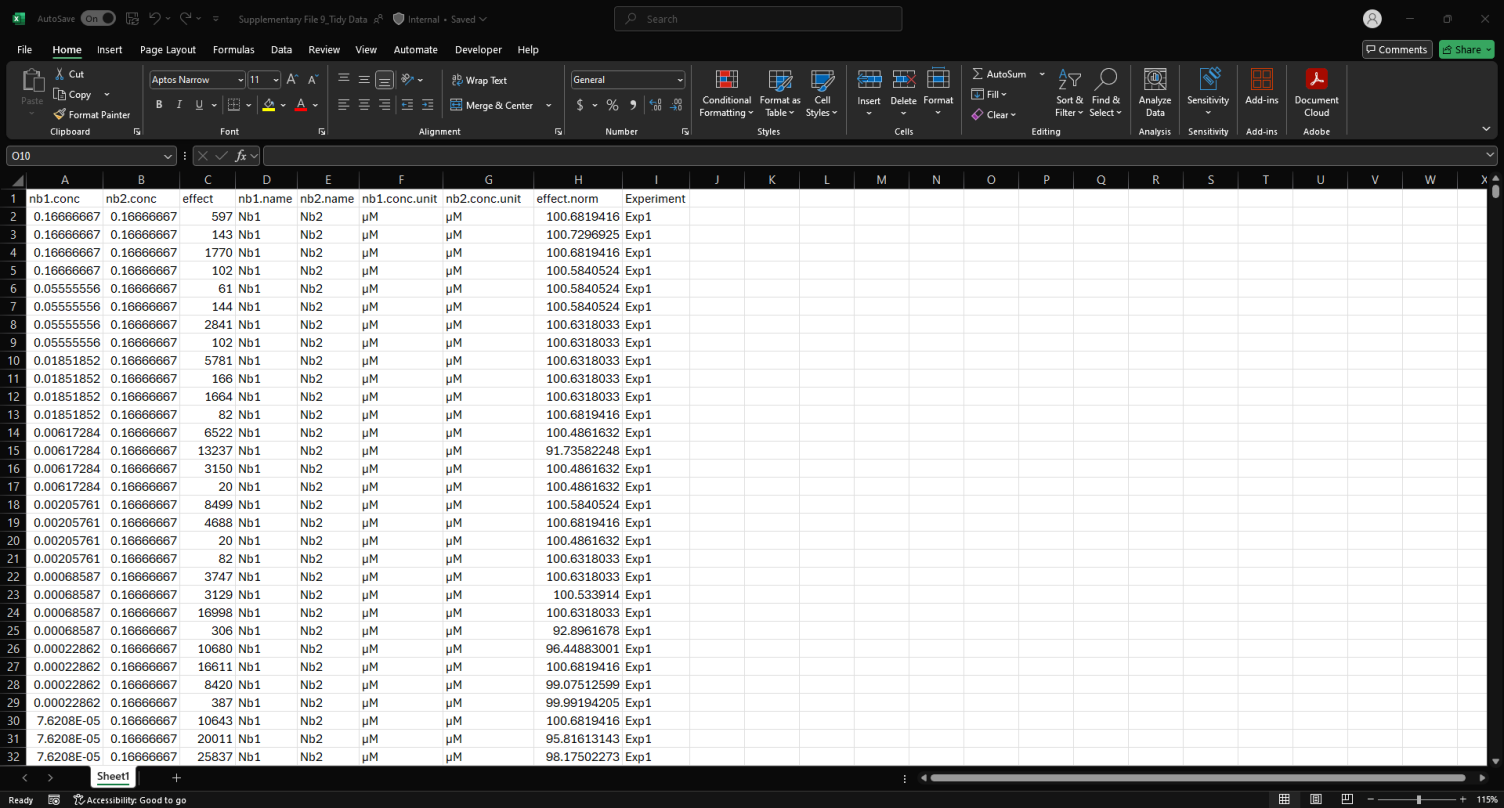
Screenshot of the Tidy Data spreadsheet (Supplementary File 9) populated with example data. This figure shows the final, normalized dataset after transferring data from the Data Reorganization sheet. Each row represents a unique nanobody pair and concentration combination, with associated normalized effect values (effect.norm) and experimental identifiers (Experiment column). This tidy format is structured for direct import into downstream Python-based synergy analysis pipelines.

**Critical:** Ensure data are in plate format prior to pasting and paste the data as values only, to preserve numeric integrity and avoid importing formatting artifacts. This operation will automatically reorganize the data into a tidy structure compatible with subsequent normalization and modeling steps.

b. Copy and paste the values from cells AA2 and AA3 into AB2 and AB3, respectively. This operation automatically populates the effect.norm column with the effect values normalized to the medium-only and virus-only controls, providing the percent neutralization (Figure 13). The Example Data sheet of Supplementary File 8 illustrates the expected appearance of the dataset after this normalization step.

c. Copy and paste cells D38:K782 from the Data Reorganization sheet into cell B1 of the Tidy Data spreadsheet (Figure 14 and Supplementary File 9). This will transfer the fully normalized and structured dataset for downstream model input and visualization.

d. Update the “Experiment” column with a unique experiment identifier to distinguish this dataset from prior synergy assays.

e. Save and close the tidy file, noting the path name to the save location for later reference.

*Note: You can combine multiple synergy experiments into a single tidy data file and use this file as a centralized repository for all synergy experiment data to streamline downstream analysis. For each new synergy assay, assign a unique experiment identifier and append the new data directly below any existing entries to maintain a continuous, structured dataset.*

2. Prepare Visual Studio Code for data analysis.

*Note: This setup only needs to be performed once per computer user account. If the required software is already installed and configured, proceed directly to step 3.*

**Figure 15.**
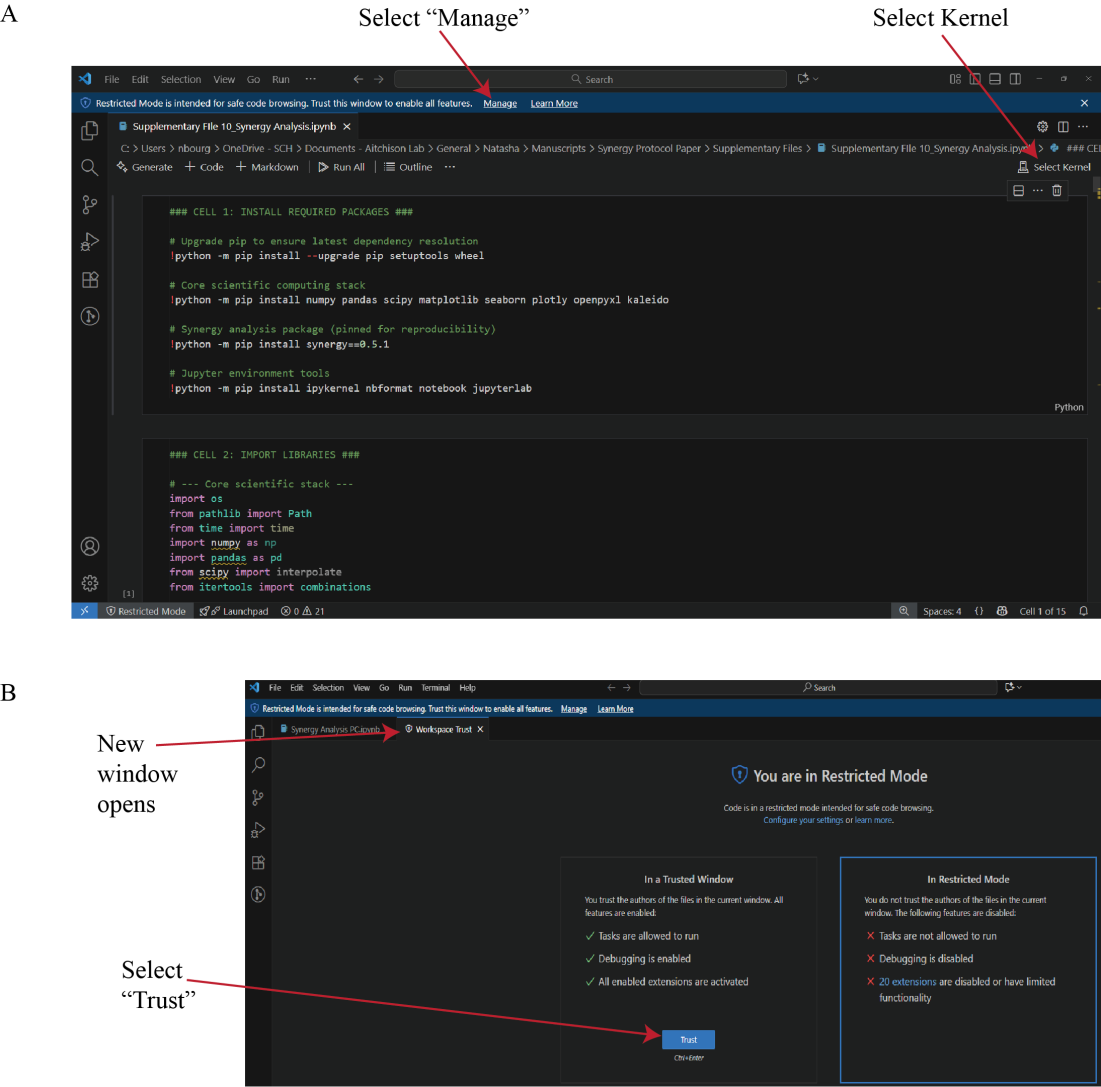
Configuration of the Synergy Analysis script in Visual Studio Code. (A) Open the Synergy Analysis file (Supplementary File 10) in Visual Studio Code. *Select Kernel* assigns the correct Python environment to the session. (B) If a *Restricted Mode* window appears, selecting *Manage* will open a new window. In the new window, select *Trust* to enable execution of all features in the workspace. This step ensures that dependent packages and kernel processes are executed under verified, reproducible conditions.

**Figure 16.**
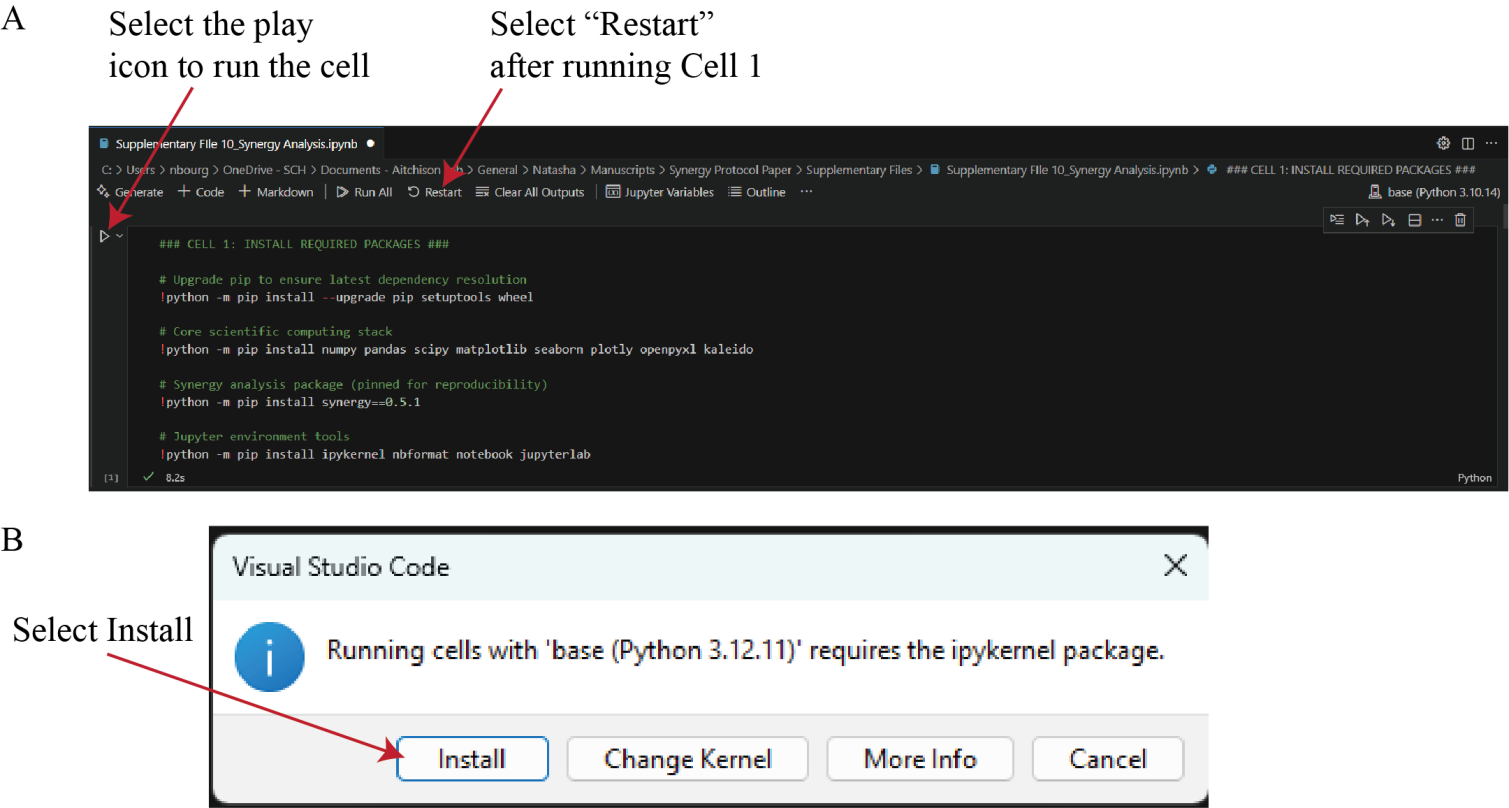
Execution of the Synergy Analysis script in Visual Studio Code. (A) After selecting the miniforge3 environment (see Data analysis step 2d), run Cell 1 by clicking the *Run Cell* (play) icon. This cell installs all required Python packages. When execution completes, select *Restart* to reload the kernel with the newly installed dependencies. (B) If a dialog box appears indicating that the ipykernel package is required, select *Install* to allow Visual Studio Code to complete the setup automatically.

a. Download and install miniforge3 from https://conda-forge.org/download/.

b. Download and install Visual Studio Code from https://code.visualstudio.com/download.

c. Open the Synergy Analysis file (Supplementary File 10) in Visual Studio Code. If prompted, select *Manage* from the blue toolbar (Figure 15), then select *Trust* in the second window that automatically opens.

d. Return to the analysis file window and select *Select Kernel* (Figure 15). From the list of available environments, choose the miniforge3 Python environment. If the environment is not displayed, refer to Troubleshooting Problem 3.

e. To execute the analysis, click the *Run Cell* (play) icon to the left of Cell 1 (Figure 16A). If a pop-up window appears prompting the installation of ipykernel, select *Install* (Figure 16B). After ipykernel finishes installing, Cell 1 will run, installing required packages.

**Caution:** Only synergy version 0.5.1 is compatible with this workflow. Newer versions of the synergy package do not work with this workflow; ensure version 0.5.1 is installed.

f. After Cell 1 completes, select *Restart* in the top toolbar (Figure 16A). Restarting the kernel reloads the Python environment, ensuring that all newly installed packages are recognized by Visual Studio Code and available for subsequent analysis steps.

**Figure 17.**
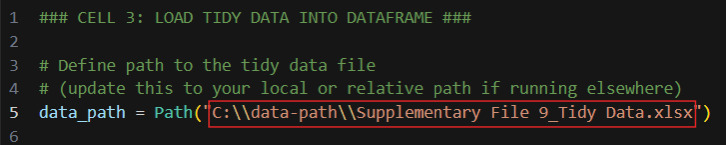
Editing Cell 3 to specify the input file path. Screenshot of Cell 3 of the Synergy Analysis file (Supplementary File 10). The red box highlights the placeholder path that must be updated with the tidy file’s path before executing the cell.

3. Use the Synergy Analysis Python file (Supplementary File 10) to analyze data.

a. If not already done, open the Synergy Analysis Python file (Supplementary File 10) in Visual Studio Code, trust the workspace, and select the Miniforge3 kernel (detailed in Data analysis steps 2c–d).

b. Run Cell 2 of the Synergy Analysis Python file (Supplementary File 10) to import required libraries. You will need to run this cell every time you open the file to ensure dependencies are loaded correctly.

c. Update the path in Cell 3 to the location of the tidy file (Figure 17); then, run the cell.

**Figure 18.**
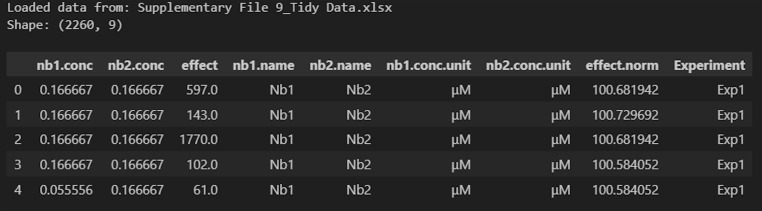
Screenshot of the output after running Cell 3 of the Synergy Analysis file (Supplementary File 10) with example data (Supplementary File 9)

**Figure 19.**
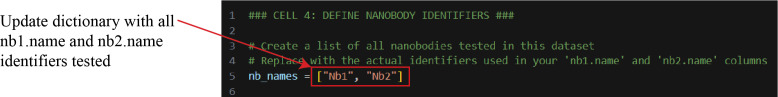
Screenshot of Cell 4 of the Synergy Analysis file (Supplementary File 10). The red box represents the text that must be updated with unique identifiers of all nanobodies you are testing.

*Note: Each time you test new nanobodies, make sure to add them to this dictionary.*

**Figure 20.**
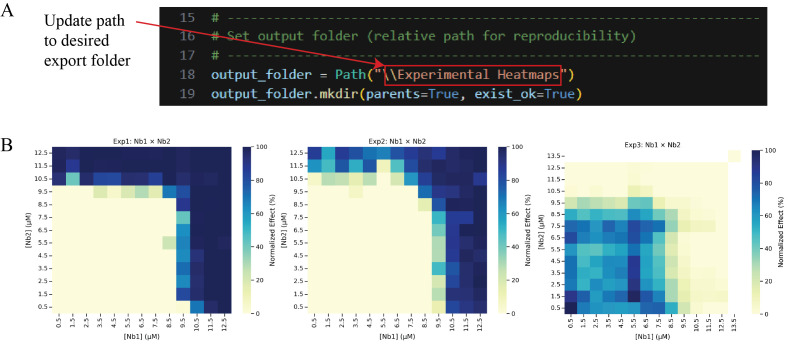
Generating and exporting experimental heatmaps. (A) Screenshot of Cell 5 of the Synergy Analysis file (Supplementary File 10) with a red box around the text that must be updated with the desired output folder path, and (B) the output files when run with example data, showing the normalized data heatmaps for individual experiments.

*Note: Data analysis steps 2g, l, n, and r also require updating the path name. Use Figure 20A as a reference for each of these steps.*

**Figure 21.**
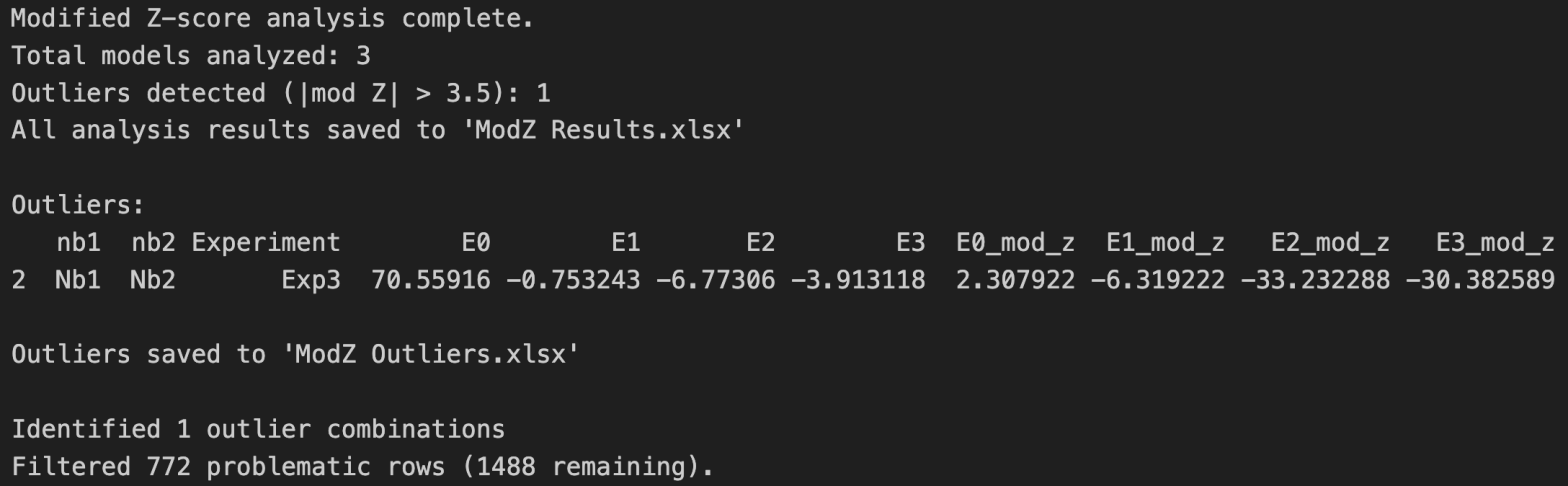
Screenshot of the output after running Cell 6 of the Synergy Analysis file (Supplementary File 10) showing a summary of the modified Z-score analysis results when example data (Supplementary File 9) is used

**Figure 22.**
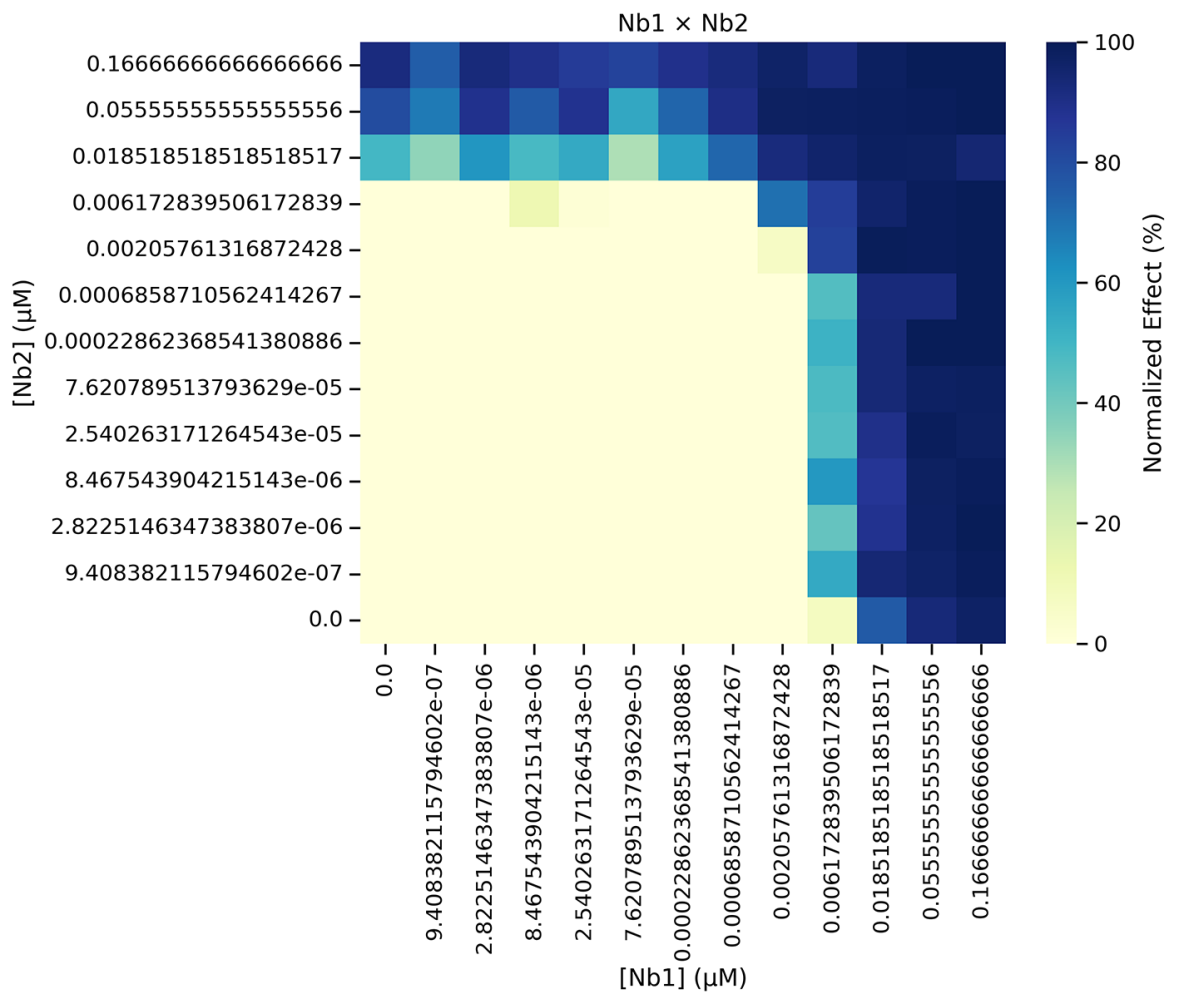
Heatmap of the normalized data that is generated from running Cell 7 of the Synergy Analysis file (Supplementary File 10) with example data, showing the aggregated results of all remaining experiments after outliers have been filtered out

Make sure you see the expected tidy file contents. Figure 18 shows the output when this cell is run with example data (Supplementary File 9).

d. Update Cell 4 with nanobody identifiers and run the cell (Figure 19). This creates a dictionary to store the identifiers of all nanobodies tested for downstream analysis.

e. Update line 18 of Cell 5 with the desired output path for normalized data heatmaps of individual experiments. Then, run the cell (Figure 20). This allows visualization across biological replicates, highlighting potential outliers. Check the contents of the output folder to ensure the heatmaps were successfully generated.

f. Run Cell 6 to perform a modified Z-score analysis on the E0, E1, E2, and E3 parameters across experiments and filter experiments that do not pass the score cutoff of |3.5| out of the data frame. This step ensures that statistical outliers are not included in subsequent analysis. An Excel file named “ModZ Results” will be generated containing the results of the analysis, and an Excel file named “ModZ Outliers” will be generated containing the results of only the outlier experiment, if an outlier is identified. Figure 21 shows the output after running this cell with example data (Supplementary File 9).

g. Update line 14 of Cell 7 with the desired output path and then run the cell. This step generates a heatmap of the normalized data across all experiments (Figure 22), rather than an individual heatmap per experiment. Check the contents of the output folder to ensure the heatmaps were successfully exported.

h. Cell 8 applies MuSyC modeling to filtered data and stores the model results in a dictionary. Broad bounds have been set to constrain the model predictions to biologically relevant values for percent neutralization data.

**Figure 23.**
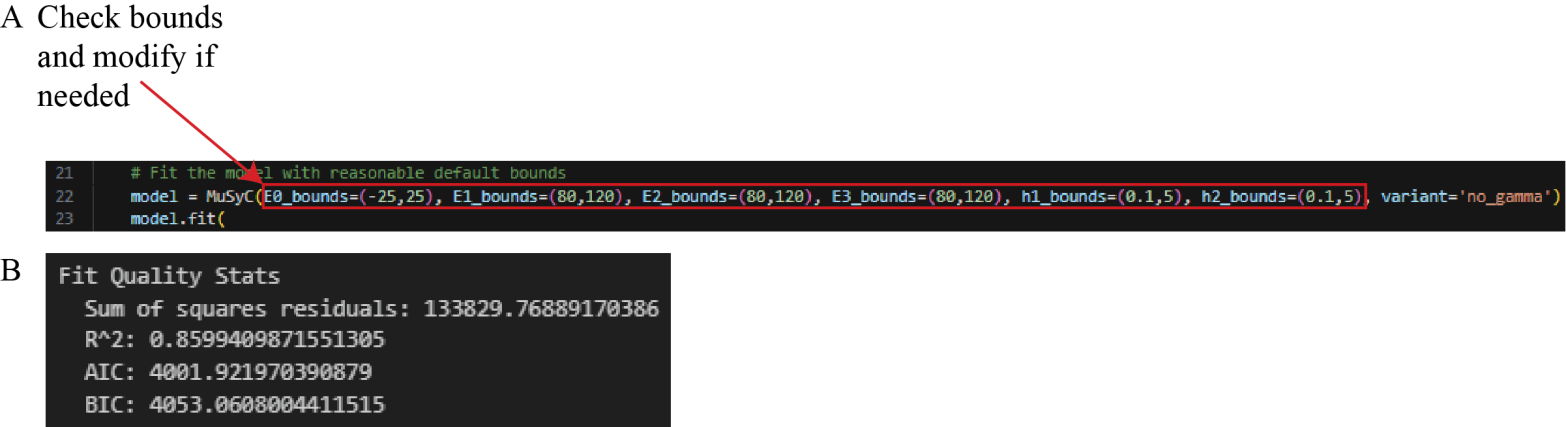
Screenshot of Cell 8 of the Synergy Analysis file (Supplementary File 10). (A) MuSyC modeling code with a red box around the text that can be modified to adjust the MuSyC model bounds. (B) The sum of square residuals, R^2^, AIC, and BIC, printed after running this cell with experimental outlier-filtered example data.

*Note: If the data does not fall within these bounds, modify these values (line 20) to set bounds corresponding to what is observed, and then run the cell (Figure 23A). For example, if nanobody 1 only achieves 60% neutralization on average, set “E1_bounds” to values encapsulating the range of percent neutralizations observed across technical replicates of the highest nanobody 1 concentration. This step also outputs the following statistics to assess the goodness of fit for the model: sum of square residuals, coefficient of determination (R^2^), Akaike Information Criterion (AIC), and Bayesian Information Criterion (BIC) (Figure 23B).*

**Figure 24.**
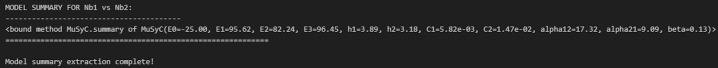
Screenshot of the output from executing Cell 9 of the Synergy Analysis file (Supplementary File 10) using example data

**Figure 25.**
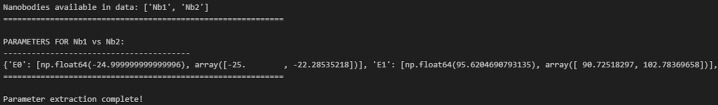
Screenshot of the output from executing Cell 10 of the Synergy Analysis file (Supplementary File 10) using example data

**Figure 26.**
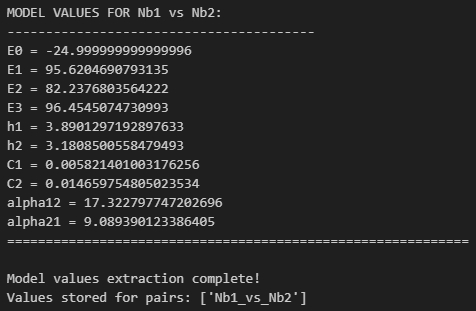
Screenshot of the output from executing Cell 11 of the Synergy Analysis file (Supplementary File 10) using example data

**Figure 27.**
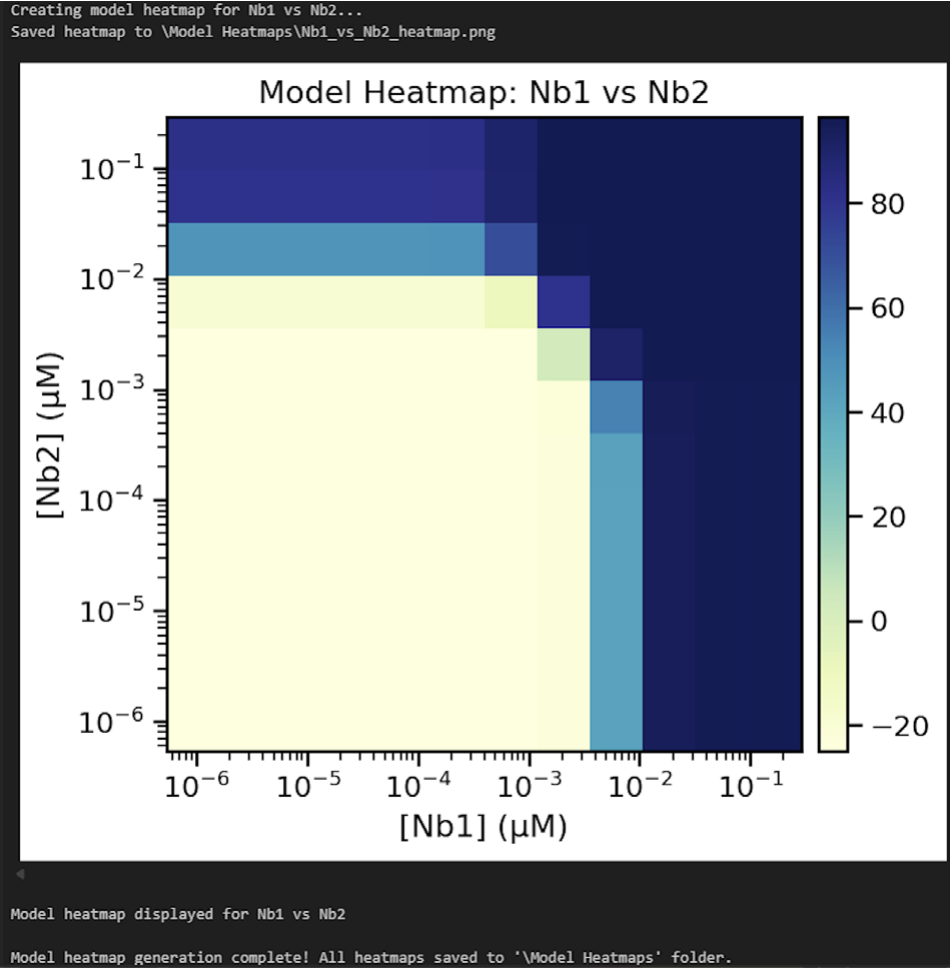
Screenshot of the output from executing Cell 12 of the Synergy Analysis file (Supplementary File 10) using example data

**Figure 28.**
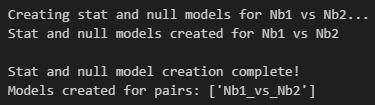
Screenshot of the output from executing Cell 13 of the Synergy Analysis file (Supplementary File 10) using example data

**Figure 29.**
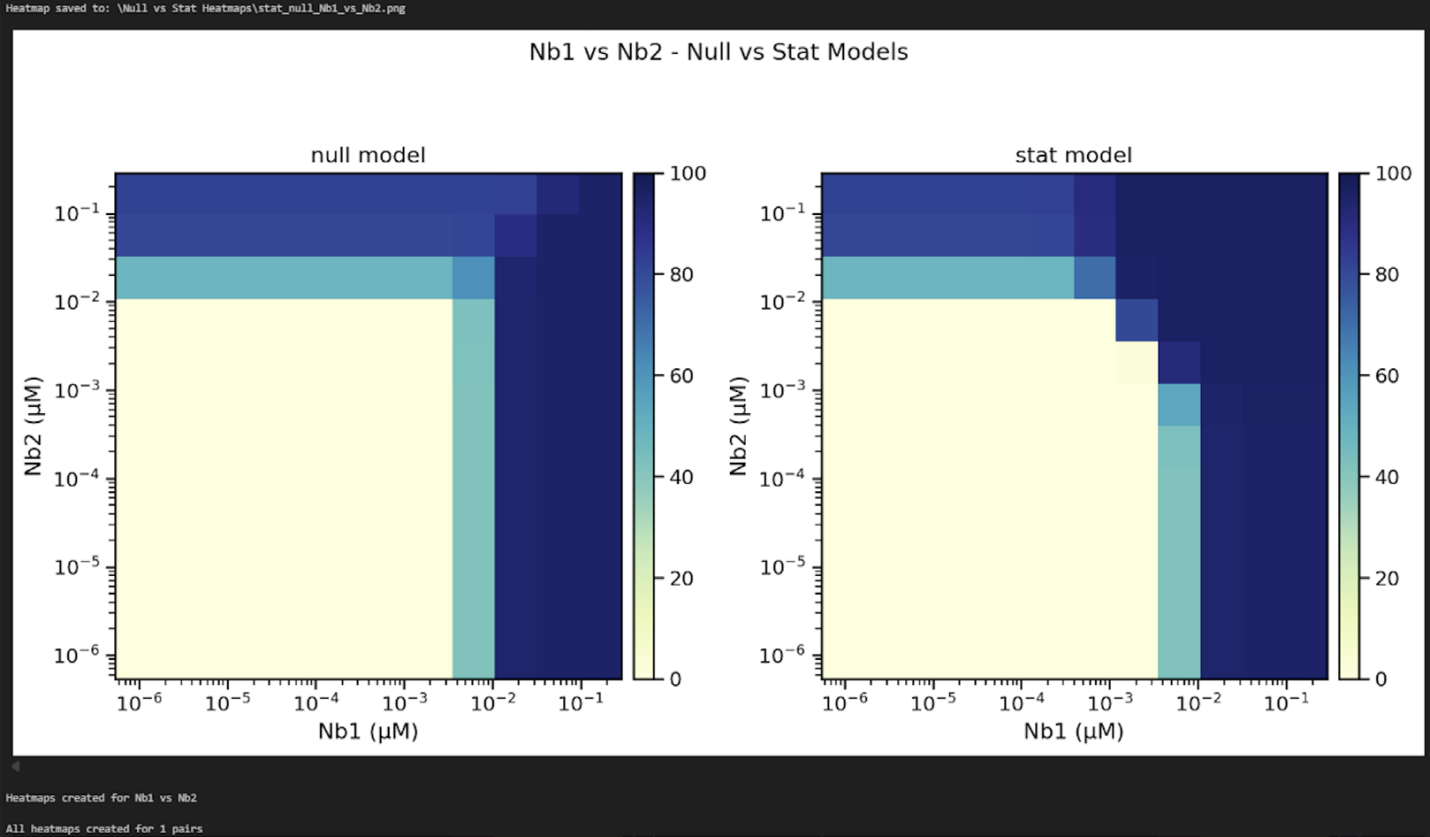
Screenshot of the output from executing Cell 14 of the Synergy Analysis file (Supplementary File 10) using example data

**Figure 30.**
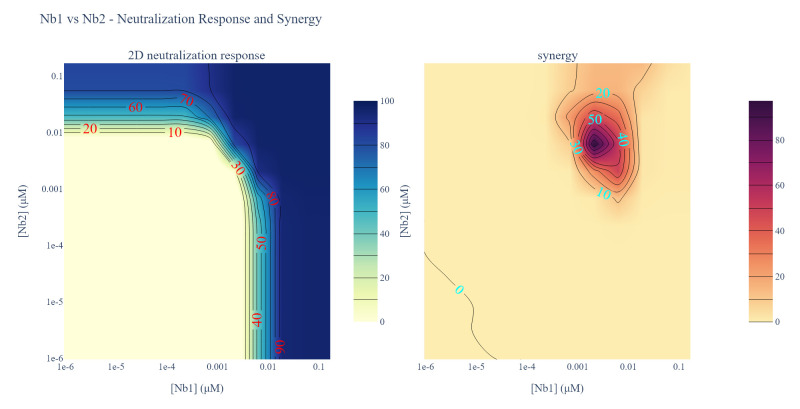
Screenshot of the output from executing Cell 15 of the Synergy Analysis file (Supplementary File 10) using example data. (Left) 2D neutralization heatmap. Lines demarcate the inhibitory concentration where X% of the virus is neutralized, and red numbers provide the percent neutralization at those points. Dark blue regions are concentrations that potently neutralize the pseudovirus, per the heatmap legend. (Right) Synergy heatmap. Lines bounding the darker purple areas demarcate regions in the heatmap where the observed neutralization is greater than additive by the indicated percentages (blue numbers). Dark purple regions are concentrations that strongly shift the potency of pseudovirus neutralization, per the heatmap legend.

i. Run Cell 9 to print a summary of the modeling results (Figure 24).

j. Run Cell 10 to print the modeling results with the 90% confidence interval (Figure 25).

k. Run Cell 11 to store model results in a dictionary for use in subsequent graphing steps (Figure 26).

l. Update line 4 of Cell 12 with the desired output folder path for the model results heatmap and then run the cell. Check the contents of the output folder to ensure the heatmaps were successfully generated (Figure 27).

m. Run Cell 13 to create and store the null model results (Figure 28).

n. Update line 32 of Cell 14 with the desired output path for the stat and null model heatmaps and then run the cell. Check the contents of the output folder to ensure the heatmaps were successfully generated (Figure 29).

o. Update line 3 of Cell 15 with the desired output path for annotated 2D neutralization (Figure 30, left) and synergy heatmaps (Figure 30, right) and then run the cell. Check the contents of the output folder to ensure the heatmaps were successfully generated.

**Result interpretation**

1. Raw data (Data analysis step 1a; Figure 12): The raw output from the luminometer shows the RLUs in each well of the 384-well cell plate. These data describe the effect of each nanobody or control on pseudoviral infection.

2. Normalized data (Data analysis step 1b; Figure 13): The raw output is normalized to the virus-only and media-only controls, such that the average value of media-only technical replicates is set to 100 and the average value of the virus-only technical replicates is set to 0; then, all data are transformed based on this range. These data describe the percent neutralization, where 100% indicates complete neutralization of infection, having the same RLU value as the media-only average, and 0% indicates no neutralization of infection, having the same RLU value as the virus-only average.

3. Normalized data heatmaps (Data analysis step 3e; Figure 20B and step 3g; Figure 22): A heatmap is generated directly from the normalized data, where dark blue indicates 100% neutralization and yellow indicates 0% neutralization.

4. Model results (Data analysis steps 3i-k; Figures 24–26): E_0_ describes the minimum effect of both nanobodies (the lowest percent neutralization observed when the concentration of both nanobodies is 0), E_1_ describes the maximum effect of nanobody 1 (the highest percent neutralization observed for nanobody 1), E_2_ describes the maximum effect of nanobody 2 (the highest percent neutralization observed for nanobody 2), and E_3_ describes the maximum effect when the nanobodies are combined. h_1_ and h_2_ describe the predicted Hill coefficients for nanobodies 1 and 2, respectively. A Hill coefficient below 1 suggests that the nanobody interferes with the binding of the other nanobody, while Hill coefficients greater than 1 suggest cooperativity between the two nanobodies; higher Hill coefficients suggest higher levels of cooperativity. C_1_ and C_2_ describe the predicted concentration at which 50% of virus is neutralized (IC50) for nanobodies 1 and 2, respectively; lower IC50s suggest greater neutralization potency. α_12_ describes the synergy coefficient that nanobody 1 affords to nanobody 2, and α_21_ describes the synergy coefficient that nanobody 2 affords to nanobody 2; higher alpha coefficients suggest greater synergy. For instance, an α_12_ of 72, as observed in the example analysis here, means that a 72-fold increase in potency of nanobody 2 is observed in the presence of nanobody 1.

5. Model heatmap (Data analysis step 3l; Figure 27): A heatmap is generated using the results of the MuSyC model on the normalized data, where dark blue indicates the maximum model-predicted value and yellow indicates the minimum model-predicted value. This graph should have similar trends to the normalized data heatmap.

6. Stat and null model heatmaps (Data analysis step 3n; Figure 29): The stat model heatmap shows the statistically significant model results, articulating where the model fit is improved when alpha is free to vary. The null model heatmap displays what the graph would look like if the nanobodies were acting independently, where all MuSyC model results were the same except for alpha, which is set to zero. Dark blue indicates 100% neutralization, and yellow indicates 0% neutralization. If synergy occurred, these graphs would look different, with the null model having a right-angle shape where the nanobody concentrations intersect.

7. 2D neutralization heatmap (Data analysis step 3o; Figure 30, left): A heatmap is generated of the pseudovirus neutralization observed by the two-dimensional serial dilution of two nanobodies. Lines demarcate the inhibitory concentration where X% of the virus is neutralized, and red numbers provide the percent neutralization at those points. Dark blue regions are concentrations that potently neutralize the pseudovirus, per the heatmap legend.

8. Synergy heatmap (Data analysis step 3o; Figure 30, right): A heatmap is generated of the neutralization synergy observed for two nanobodies. The lines bounding the darker purple areas demarcate regions in the heatmap where the observed neutralization is greater than additive by the indicated percentages (blue numbers). Dark purple regions are concentrations that strongly shift the potency of pseudovirus neutralization, per the heatmap legend.

## Validation of protocol

This protocol has been used and validated in the following research articles:

• Mast et al. [6]. Highly synergistic combinations of nanobodies that target SARS-CoV-2 and are resistant to escape (Figure 8, Table 9).

• Ketaren et al. [7]. Nanobody repertoire generated against the spike protein of ancestral SARS-CoV-2 remains efficacious against the rapidly evolving virus (Figure 7).

## General notes and troubleshooting

**General notes**

1. Complete Procedure steps B3–6 for one nanobody pair at a time. If testing multiple nanobody pairs in one day, complete steps B3–6 for a given pair before beginning the next pair to avoid exceeding 1 h of incubation period or evaporation of small volumes in the 96-well deep-well plate.

2. Inactivate all materials exposed to (pseudo)virus. Ensure all materials that encounter the virus are treated with 10% bleach for at least 10 min before disposing. Follow local Environmental Health and Safety regulations.

3. Control luminescent signal intensity. A strong luminescent signal can cause light spillover into nearby wells. Use a volume of virus stock that achieves ~100,000 RLUs and ensure the luminescent detector is as close to the plate top as possible to reduce technical noise.

4. Use the modified Steady-Glo^®^ handling steps. The instructions provided for reagent preparation and assay procedure in the Steady-Glo^®^ technical manual have led to inconsistencies in RLU detection. Follow Procedure steps C1–2 for the best performance of this reagent.

5. Use validated Gilson labware configurations. The Gilson protocols we provide here have been optimized for the specific labware reported in the Laboratory Supplies section. The use of different labware may require alterations in these protocols, which can be done in the Protocol Builder software. We provide the Protocol Builder formatted files for all protocols, as well as a guide for using the software, to accommodate this (Supplementary Files 11–16). See step 4 of the Protocol Builder Instructions document (Supplementary File 11) for an overview of the protocol parameters that are critical to optimize before running these Gilson protocols.

6. Maximize deep-well plate usage. Only half of the deep-well plate is used for each synergy assay. Rotate the plate 180º to use the other side for another synergy assay and keep the plate sterile in between experiments.

7. Verify plate seating. The risers in the Gilson have a spring mechanism to keep the plates in place. If plates are not properly nested into the riser, they may move during a protocol or interfere with the pipette head path. Make sure plates are flush with the riser.

8. Ensure that the tip waste catch bucket for the Gilson is empty prior to running protocols. Overfilled tips can interfere with the platform’s movement and cause errors in the Gilson.

9. Proper storage of biological materials and reagents is essential. Do not use materials after the manufacturer-provided expiration date. Nanobodies, pseudovirus, and cell lines are stable for years at the indicated storage temperature. Use medium with a pH indicator (e.g., phenol red) to prevent the use of acidified media, which causes cellular stress. Keep pseudovirus in small volume aliquots to prevent repeated freeze-thaw cycles, which reduce viability.

**Troubleshooting**

**Problem 1:** Inconsistent aspiration or dispensing volumes occur during automated liquid handling.

Possible cause: Slight differences in labware or Gilson risers may require adjustment of the immersion depth of the pipette tips as they go into the wells.

Solution: Adjust the Z-offset setting in the Protocol Builder software such that the pipette tips are not going too far down, causing contact with the bottom of the well, or not going far down enough such that contact with liquid is not maintained.

**Problem 2:** Miniforge3 environment does not automatically show up as an option when selecting kernel (Data analysis step 2e, Figure 16).

Possible cause: Miniforge3 install is not being registered by VS Code.

Solution: In VS Code, press Ctrl + Shift + P on the keyboard. Search for “Python: Select Interpreter” and then select it. Select *Enter interpreter path* and then *Find…*. Use the file explorer to select the python.exe file in the Miniforge3 folder that was generated during the Miniforge3 install (Data analysis step 2a). Select *Select Kernel* and then select *MiniForge3 environment*.

**Problem 3:** Dose-response data deviate from single-nanobody reference experiments.

Possible cause 1): Plates were put into the Gilson in an incorrect orientation.

Solution 1): Ensure plates are placed in the Gilson risers with A1 in the upper left corner of the element.

Possible cause 2): Endotoxin contamination of the nanobody batch. Residual bacterial endotoxin can cause nonspecific cytotoxicity or alter cell metabolism, producing flattened or erratic dose–response profiles.

Solution 2): Test the nanobody stock using a LAL assay or equivalent endotoxin detection kit. If levels exceed acceptable limits (≥0.1 EU/μg protein), repurify using polymyxin B resin or detergent-based endotoxin removal before repeating the assay. Maintain sterile technique during purification and storage.

Possible cause 3): Nanobody precipitation. Protein instability or sub-optimal storage can cause visible precipitate formation, reducing the effective nanobody concentration in solution.

Solution 3): Examine each stock for turbidity or visible particulates. Centrifuge at >10,000× *g* for 5 min to clarify, then determine soluble protein concentration by A_280_ or BCA assay. Store nanobody stocks at 4 °C (short-term) or -80 °C (long-term). Avoid repeated freeze-thaw cycles of nanobodies.

## Supplementary information

The following supporting information can be downloaded here:

1. Supplementary File 1. Cell Plating.sqlite

2. Supplementary File 2. Deep-Well Plate Map.xlsx

3. Supplementary File 3. Nanobody Virus Mix.sqlite

4. Supplementary File 4. 96-well V-bottom Plates Map.xlsx

5. Supplementary File 5. Transfer to Cells.sqlite

6. Supplementary File 6. Cell Plates Map.xlsx

7. Supplementary File 7. Steady Glo.sqlite

8. Supplementary File 8. Synergy Data Reorganization.xlsx

9. Supplementary File 9. Tidy Data.xlsx

10. Supplementary File 10. Synergy Analysis.ipynb

11. Supplementary File 11. Protocol Builder Instructions.docx

12. Supplementary File 12. Cheat Sheet Gilson.docx

13. Supplementary File 13. Cell Plating.pedb

14. Supplementary File 14. Nanobody Virus Mix.pedb

15. Supplementary File 15. Transfer to Cells.pedb

16. Supplementary File 16. Steady Glo.pedb
